# Sex differences in immune protection in mice conferred by heterologous vaccines for pneumonic plague

**DOI:** 10.3389/fimmu.2024.1397579

**Published:** 2024-05-21

**Authors:** Michael L. Davies, Sergei S. Biryukov, Nathaniel O. Rill, Christopher P. Klimko, Melissa Hunter, Jennifer L. Dankmeyer, Jeremy A. Miller, Jennifer L. Shoe, Kevin D. Mlynek, Yuli Talyansky, Ronald G. Toothman, Ju Qiu, Joel A. Bozue, Christopher K. Cote

**Affiliations:** ^1^ Bacteriology Division, United States Army Medical Research Institute of Infectious Diseases, Fort Detrick, Frederick, MD, United States; ^2^ Regulated Research Administration: Biostatistics Division, United States Army Medical Research Institute of Infectious Diseases, Fort Detrick, Frederick, MD, United States

**Keywords:** live attenuated vaccine, prime-boost immunization, plague, *Yersinia pestis*, mice, pneumonic, F1-V, sex differences

## Abstract

**Background:**

*Yersinia pestis* is the etiological agent of plague, which can manifest as bubonic, septicemic, and/or pneumonic disease. Plague is a severe and rapidly progressing illness that can only be successfully treated with antibiotics initiated early after infection. There are no FDA-approved vaccines for plague, and some vaccine candidates may be less effective against pneumonic plague than bubonic plague. *Y. pestis* is not known to impact males and females differently in mechanisms of pathogenesis or severity of infection. However, one previous study reported sex-biased vaccine effectiveness after intranasal *Y. pestis* challenge. As part of developing a safe and effective vaccine, it is essential that potential sex differences are characterized.

**Methods:**

In this study we evaluated novel vaccines in male and female BALB/c mice using a heterologous prime-boost approach and monitored survival, bacterial load in organs, and immunological correlates. Our vaccine strategy consisted of two subcutaneous immunizations, followed by challenge with aerosolized virulent nonencapsulated *Y. pestis*. Mice were immunized with a combination of live *Y. pestis pgm-* pPst*-*Δ*caf1*, live *Y. pestis pgm-* pPst*-*Δ*caf1*/Δ*yopD*, or recombinant F1-V (rF1-V) combined with adjuvants.

**Results:**

The most effective vaccine regimen was initial priming with rF1-V, followed by boost with either of the live attenuated strains. However, this and other strategies were more protective in female mice. Males had higher bacterial burden and differing patterns of cytokine expression and serum antibody titers. Male mice did not demonstrate synergy between vaccination and antibiotic treatment as repeatedly observed in female mice.

**Conclusions:**

This study provides new knowledge about heterologous vaccine strategies, sex differences in plague-vaccine efficacy, and the immunological factors that differ between male and female mice.

## 1 Introduction

*Yersinia pestis*, the plague bacterium, has been a public health hazard for centuries (1–3). The predominant mode of infection in historical pandemics, bubonic plague transmitted by flea or louse vectors, is now treatable with antibiotics if treatment is initiated early after infection, and plague outbreaks are generally rare and localized. However, bubonic plague can develop into septicemic plague and spread to the lungs as a secondary infection resulting in pneumonic plague. Septicemic and pneumonic plague are rapidly progressing diseases that are usually fatal if not treated early after the onset of symptoms (4, 5). Pneumonic plague presents a distinct public health and biodefense threat, because in this form the disease can be spread via aerosol. Several reports of person-to-person pneumonic spread have come from Asia, Africa, and South America in recent years (6–8), including outbreaks of over 100 pneumonic cases in two nations where *Y. pestis* is highly endemic, Madagascar and Democratic Republic of the Congo (9, 10).

Numerous historical attempts have been made to use *Y. pestis* as a bioweapon due to its lethality and ability to spread via aerosol, lending credence to its designation as a Tier 1 select agent by the United States Department of Health and Human Services (11). Early vaccination approaches shifted from heat-killed bacteria to live attenuated vaccines (LAVs) upon observation that only the latter could protect against inhalation challenge (12, 13) and induce effective cellular immunity rather than a predominantly humoral response (14, 15). This was followed by LAVs with targeted deletions in virulence factors, and subunit vaccines primarily designed to raise immunity against the dominant antigens F1 (capsule antigen Caf1, coating most strains of *Y. pestis*) and V (type III injectisome cap protein LcrV, essential for virulence and secretion of virulence factors called Yersinia outer proteins [Yops]) (16–18). Subunit vaccines consisting of F1 and V complexed with adjuvants have been highly protective in several animal models of pneumonic plague (19–28). However, possible alteration of bacteria, known heterogeneity of the V protein in other *Yersinia* species, and existence of virulent F1-negative strains of *Y. pestis* (22, 29, 30), mean that broader immunity in addition to these proteins is necessary to combat a wide range of bacterial isolates.

The common progression of plague after exposure to aerosolized *Y. pestis*, in species ranging from humans and non-human primates to mice, is biphasic (11, 31, 32). An initial “preinflammatory” phase is marked by bacterial replication in the absence of severe symptoms or immune response in the lungs, due to several immune modulating proteins produced by *Y. pestis*. After a period of time, about 36 h in mice (dependent upon the strain of *Y. pestis* and inhaled dose), the “proinflammatory” phase begins, with a systemic immune response, uncontrolled release of inflammatory mediators in the lungs, and delayed recruitment of neutrophils to the lungs. Many antibiotics are effective treatments for pneumonic plague, but only if treatment is initiated within a day or two of the onset of symptoms (11, 33). In addition to preventing disease, a goal of vaccine development is to extend the window of time in which antibiotic treatment (or other therapeutic strategies) will rescue pneumonic plague patients. In recent studies, our laboratory has been pursuing a vaccination strategy focusing on combatting potential infection with F1-negative strains. These efforts include characterizing vaccines that induce immunity against not just F1-positive but also F1-negative strains and demonstrating synergy with post-exposure antibiotic treatments in vaccinated individuals (34).

The virulent *Y. pestis* strains used in this study are Colorado 92 (CO92), which has an intact F1 capsule (35), and the F1-negative strain C12 (36). We have made a C12 strain with a deletion mutation in *yscN*, which encodes the ATPase needed for type 3 secretion system (T3SS) function (37). The lethality of the C12 Δ*yscN* strain was attenuated by over 6 orders of magnitude, similar to the effect of deleting *yscN* in CO92 (38, 39). In a sensitive BALB/c mouse model, a homologous two-dose vaccine strategy combining CO92 Δ*yscN* and C12 Δ*yscN* (“Combo Δ*yscN*”) was effective against lethal bubonic plague caused by either *Y. pestis* CO92 or C12, but only against pneumonic plague caused by *Y. pestis* CO92 (34, 39). Survival was improved by using a heterologous two-dose vaccine regimen: one dose of LAV followed by one dose of recombinant F1-V (rF1-V) combined with adjuvants CpG (ODN 2006) and Alhydrogel (Alh) (34). Survival after challenge and control of bacterial replication in tissues were highly correlated with enhanced anti-rF1-V serum antibody titers (34).

The present study aims to further establish the efficacy of heterologous vaccine strategies against lethal F1-negative pneumonic plague. Initially, we continued the heterologous approach combining “Combo Δ*yscN*” LAV with subunit vaccines based on rF1-V. We then pursued a new strategy for a safer live attenuated F1-negative strain, by deleting *caf1* and/or *yopD* from the already-attenuated strain CO92 *pgm*- pPst-. This strain has a 102kb deletion in the pigmentation locus (*pgm*) required for iron acquisition and storage, resulting in well-established attenuation (40–42), and is also cured of the pPst plasmid (also named pPCP1, pPla) encoding the plasminogen activator enzyme that enhances fibrinolysis and bacterial dissemination *in vivo*. The curing of pPst potentially enhances the safety profile, in particular for individuals with undiagnosed hemochromatosis (43, 44). Deleting *caf1* produces a capsule-negative *pgm*- pPst- strain. In addition, deleting *yopD* enhances expression of Yersinia Outer Proteins (Yops) in the bacterium, and blocks the bacteria from injecting Yops into host cells (45, 46). The protein profile expressed by this mutant may enrich antigens for protective immunity in the absence of the highly immunogenic capsule that appears to dominate the humoral immune response. These multiple stable deletion mutations result in an attenuated LAV that could be potentially safe for advanced development and are excluded from select-agent regulations.

Here we investigate the protective efficacy and immune correlates of these new LAV strains as part of a heterologous vaccine strategy. In addition, we immunized both male and female mice to discern potential sex differences in the immunity induced by *Y. pestis* vaccines. Our data from comparisons of male and female mice build on those reported by Bowen et al. (47) in which the authors described significant challenges protecting male relative to female mice from pneumonic plague using the rF1-V + Alhydrogel vaccine.

## 2 Results

### 2.1 Heterologous vaccination using *Y. pestis* Δ*yscN* live attenuated vaccine strains followed by a protein subunit booster in female and male BALB/c mice

Our recent work described successful vaccination strategies using the Δ*yscN* vaccine strains that protected female BALB/c mice from pneumonic plague initiated by aerosolized *Y. pestis* CO92 (encapsulated) and *Y. pestis* C12 (nonencapsulated) strains (34, 39). Here, we performed follow-on studies to compare the efficacy of these vaccines in male BALB/c mice. Groups of male BALB/c mice were given an initial (“prime”) vaccine injection with a combination of CO92 Δ*yscN* and C12 Δ*yscN* (“Combo Δ*yscN*”), followed three weeks later by a “boost” injection with either Combo Δ*yscN* or a subunit vaccine. The subunit vaccine was rF1-V, mixed with Alhydrogel, and CpG as an additional adjuvant (**Table 1**, experiment A). Sham immunization was an injection of 0.2 mL PBS for both prime and boost, as in all subsequent experiments. In descriptions of these vaccine regimens, in text and figure legends, “P” indicates the prime injection and “B” indicates the boost injection.

**Table 1:** Vaccine experiment details.

Experiment	Groups	Vaccine Prime	Prime-boost interval	Vaccine Boost	3 days pre-challenge samples	Boost-challenge interval	Aerosol challenge* dose	Streptomycin **	3 days post-challenge samples***
A	Male	PBS (sham)	21 d	PBS (sham)	nd	31 d	1.1 x 10^5^	nd	*n*=6
	Male	Combo Δ*yscN*		Combo Δ*yscN*	nd		1.1 x 10^5^	nd	*n*=6
	Male	Combo Δ*yscN*		rF1-V****	nd		1.1 x 10^5^	nd	*n*=6
B	Female	PBS (sham)	21 d	PBS (sham)	*n*=4	28 d	4.7 x 10^5^	nd	*n*=4
	Male	PBS (sham)		PBS (sham)	*n*=4		5.7 x 10^5^	nd	*n*=4
	Female	rF1-V		Δ*caf1*	*n*=4		4.7 x 10^5^	nd	*n*=3
	Male	rF1-V		Δ*caf1*	*n*=4		5.7 x 10^5^	nd	*n*=4
	Female	Δ*caf1*		rF1-V	*n*=3		4.7 x 10^5^	nd	*n*=3
	Male	Δ*caf1*		rF1-V	*n*=4		5.7 x 10^5^	nd	*n*=4
C	Female	PBS (sham)	21 d	PBS (sham)	*n*=4	29 d	2.0 x 10^5^	nd	*n*=4
	Male	PBS (sham)		PBS (sham)	*n*=4		2.4 x 10^5^	nd	*n*=4
	Female	rF1-V		Δ*yopD*/Δ*caf1*	*n*=4		2.0 x 10^5^	nd	*n*=4
	Male	rF1-V		Δ*yopD*/Δ*caf1*	*n*=4		2.4 x 10^5^	nd	*n*=4
	Female	Δ*yopD*/Δ*caf1*		rF1-V	*n*=4		2.0 x 10^5^	nd	*n*=4
	Male	Δ*yopD*/Δ*caf1*		rF1-V	*n*=4		2.4 x 10^5^	nd	*n*=4
D	Female	PBS (sham)	28 d	PBS (sham)	nd	28 d	6.7 x 10^5^	nd	nd
	Male	PBS (sham)		PBS (sham)	nd		7.7 x 10^5^	nd	nd
	Female	PBS (sham)		PBS (sham)	nd		6.7 x 10^5^	20 mg/kg	nd
	Male	PBS (sham)		PBS (sham)	nd		7.7 x 10^5^	20 mg/kg	nd
	Female	rF1-V		Δ*caf1*	nd		6.7 x 10^5^	nd	nd
	Male	rF1-V		Δ*caf1*	nd		7.7 x 10^5^	nd	nd
	Female	Δ*caf1*		rF1-V	nd		6.7 x 10^5^	nd	nd
	Male	Δ*caf1*		rF1-V	nd		7.7 x 10^5^	nd	nd
	Female	Δ*caf1*		rF1-V	nd		6.7 x 10^5^	20 mg/kg	nd
	Male	Δ*caf1*		rF1-V	nd		7.7 x 10^5^	20 mg/kg	nd
	Female	Δ*yopD*/Δ*caf1*		rF1-V	nd		6.7 x 10^5^	nd	nd
	Male	Δ*yopD*/Δ*caf1*		rF1-V	nd		7.7 x 10^5^	nd	nd
	Female	Δ*yopD*/Δ*caf1*		rF1-V	nd		6.7 x 10^5^	20 mg/kg	nd
	Male	Δ*yopD*/Δ*caf1*		rF1-V	nd		7.7 x 10^5^	20 mg/kg	nd

*After challenge, in all cases mice were followed for 21 days to assess survival, with *n*=10 in all groups except where indicated.

**Every 6 hours, starting 60 hours post-challenge.

***In Experiments A-C, in addition to the mice followed for survival, a separate group of mice were euthanized for assessment at 3 dpi.

****In all cases, rF1-V indicates 2 µg rF1-V complexed with CpG and Alhydrogel.

**Figure 1:**
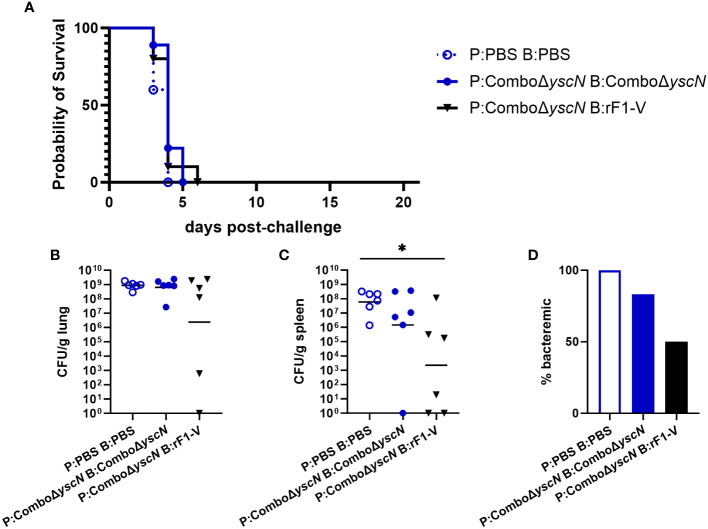
Survival and bacterial burden 3 dpi of male BALB/c mice immunized with Combo Δ*yscN* regimens and challenged with aerosolized *Y. pestis* C12. **(A)** Male mice (*n* = 10/group) were vaccinated with the regimens in the legend, with 21 days between injections, then challenged with aerosolized C12 (1.1 x 10^5^ CFU) at 31 d post-boost. Clinical progression was monitored daily for 21 days. **(B-D)** Male mice given the same vaccine regimens and challenge were euthanized 3 days post-challenge. Lung homogenates **(B)**, spleen homogenates **(C)**, and whole blood **(D)** were plated to quantify bacterial burden. Graphs show each data point and lines representing geometric means. *p < 0.05 in Mann-Whitney test.

Four weeks post-boost, male mice were challenged with aerosolized *Y. pestis* C12 (calculated inhaled dose 1.1x10^5^ CFU). 10 mice in each group were followed for survival, and 6 were euthanized at 3 dpi (days post-infection) for measurement of bacterial burden. All male mice succumbed to infection by 6 dpi (**Figure 1A**). In previous studies using the same vaccines in female mice (34), survival was 90% with the P:Combo Δ*yscN* B:rF1-V strategy, and 0% in females given P:Combo Δ*yscN* B:Combo Δ*yscN* or sham (P: PBS B: PBS). The increased survival in females, despite a higher calculated exposure dose (7.9x10^5^ CFU) in the female experiment, led us to further characterize sex differences in this infection model.

We performed an LD_50_ estimation using 7–9-week-old naïve male BALB/c mice. As shown in **Supplementary Figure 1**, the LD_50_ value for *Y. pestis* C12 was between 1.6x10^4^ and 3.2x10^5^ inhaled CFU. Given that our calculated LD_50_ value for female BALB/c mice is approximately 7.7x10^4^ CFU (22), the overall virulence of *Y. pestis* C12 in male mice does not appear to differ from female mice and is likely not the source of the differences in vaccine efficacy.

### 2.2 Bacterial burden in male mice challenged after immunization with Combo Δ*yscN* regimens

In each group above, 6 mice were euthanized for sample collection at 3 dpi. We find 3 dpi to be a timepoint where *Y. pestis* replication in susceptible mice is near peak in both lungs and secondary tissues and sham-vaccinated mice are still available for a direct comparison of samples (34, 48, 49). Blood and organ homogenates were assessed by using serial dilutions plated on SBA to enumerate CFU. All animals but one had detectable bacteria in the lungs, with no significant difference between vaccinated groups and sham (**Figure 1B**). However, in the spleen, those given P:Combo Δ*yscN* B:rF1-V had significantly less bacteria detected (**Figure 1C**), indicating that systemic spread of *Y. pestis* was reduced despite no protection from lethal disease. Whereas 100% of the control mice had bacteria in their blood, only 50% of the mice receiving heterologous vaccines were bacteremic (**Figure 1D**). No significant reductions in bacterial burden were seen in male mice given two doses of Combo Δ*yscN* (**Figures 1B–D**).

### 2.3 Heterologous vaccination using rF1-V in combination with capsule-negative strains derived from *pgm*- pPst- attenuated *Y. pestis*

Although the Δ*yscN* strains are considerably attenuated (in subcutaneous challenge they have an LD_50_ over 10^6^-fold greater than parent CO92 or C12 strains) (39), the attenuation is entirely due to the deletion of the *yscN* gene and they do not meet the requirements for excluded status. Hence, these vaccine strains must be handled under Biosafety Level 3 (BSL3) conditions and retain federal select-agent designation and the associated regulatory burden. Therefore, we decided to test another strategy to create a vaccine strain that is both attenuated and nonencapsulated. The attenuated strain used to create new mutants was the established CO92 *pgm*- pPst- mutant, which is excluded from the select agent list based on established criteria (https://www.selectagents.gov/sat/exclusions/hhs.htm) (42). In addition, based on historical data, encapsulated LAVs induce a robust anti-F1 response that appears to dominate the protective humoral arm of the immune response, likely redirecting antibody responses from other potentially protective targets such as Yops or V antigen. To alleviate possible interference of F1 in the induction of the immune response to other antigens, a *caf1* deletion mutant on CO92 *pgm-* pPst- background (Δ*caf1*) was constructed. Furthermore, a *yopD* mutation was shown to upregulate expression of various T3SS associated genes, including effector Yops and V (45). To further promote the induction of an immune response to more diverse protective antigens that may be naturally present at below immune-activation threshold levels, a *yopD* mutation (Δ*yopD*/Δ*caf1*) was also constructed in the *pgm*- pPst- strain.

To test the protective efficacy of these strains as LAVs, BALB/c mice were immunized via heterologous prime-boost vaccine regimens. For half of the immunized animals, the prime was with a LAV (either Δ*caf1* or Δ*yopD*/Δ*caf1*), followed by boost with rF1-V. For the other half, the prime was with rF1-V, and the boost was with Δ*caf1* or Δ*yopD*/Δ*caf1* LAV (**Table 1**, experiments B and C). Equal numbers of age-matched males and females were used. Males and females were exposed simultaneously to the same aerosolized bacteria; however, the males were estimated to have inhaled about 15-20% more bacteria, based on differences in body mass and estimated lung volumes (50, 51).

**Figure 2:**
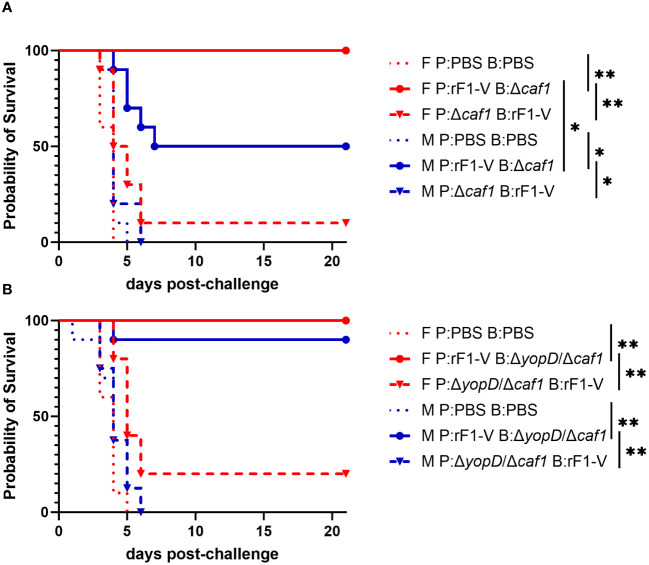
Survival of BALB/c mice immunized with Δ*caf1* or Δ*yopD*/Δ*caf1* regimens and challenged with aerosolized *Y. pestis* C12. **(A)** Female (F) and male (M) mice (*n* = 10/group) were vaccinated with the Δ*caf1* regimens in the legend, with 21 days between injections, then challenged with C12 (4.7 x 10^5^ CFU for females, 5.7 x 10^5^ CFU for males) at 28 d post-boost. **(B)** Female (F) and male (M) mice (*n* = 10/group) were vaccinated with the Δ*yopD*/Δ*caf1* regimens in the legend, with 21 days between injections, then challenged with C12 (2.0 x 10^5^ CFU for females, 2.4 x 10^5^ CFU for males) at 29 d post-boost. Clinical progression was monitored daily for 21 days. *p < 0.05, **p < 0.01 in pairwise comparison of groups by probability of survival (%) using a Fisher exact test.

Only 10% of female and 0% male mice immunized with P:Δ*caf1* B:rF1-V were protected against aerosol challenge with ~6-7 LD_50_ of strain C12. However, when the order was reversed and mice were immunized with P:rF1-V B:Δ*caf1*, there was significantly higher survival for both sexes. Furthermore, there was significantly higher survival in females than males given this vaccine regimen (100% for females, 50% for males) (**Figure 2A**). The importance of giving the rF1-V vaccine first was also seen using Δ*yopD*/Δ*caf1* LAV. 100% of females and 90% of males survived aerosol challenge when immunized with P:rF1-V B:Δ*yopD*/Δ*caf1*, compared to 20% and 0% when the order was reversed (**Figure 2B**). Males and females had similarly high survival in this experiment, likely related to the lower estimated inhaled dose in this iteration.

### 2.4 Bacterial burden in mice challenged with *Y. pestis* C12 after heterologous vaccination with rF1-V and Δ*caf1* or Δ*yopD*/Δ*caf1* LAV

**Figure 3:**
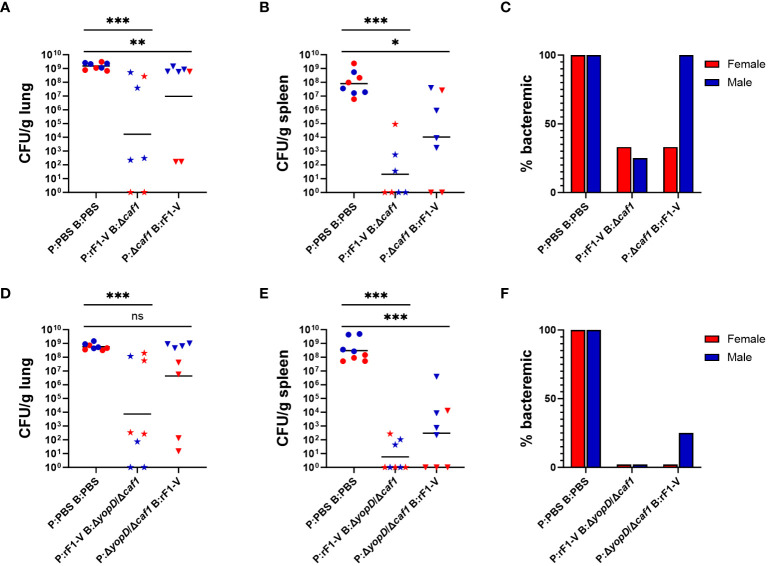
Bacterial burden of BALB/c mice immunized with Δ*caf1* or Δ*yopD*/Δ*caf1* regimens and challenged with aerosolized *Y. pestis* C12. Female (red) and male (blue) mice were vaccinated with Δ*caf1* regimens **(A–C)** or Δ*yopD*/Δ*caf1* regimens **(D–F)** as in **Figure 2**. Three dpi, mice were euthanized and lung homogenates **(A, D)**, spleen homogenates **(B, E)**, and whole blood **(C, F)** were plated to quantify bacterial burden. Graphs show each data point and lines representing geometric means. **p < 0.01, ***p < 0.001 in Mann-Whitney test. ns, not significant.

Mice were euthanized at 3 dpi and bacterial burdens in lungs, spleen and blood were quantified as described above. Among mice immunized with Δ*caf1* regimens, those that received P:rF1-V B:Δ*caf1*, which was 100% protective in females and 50% in males, had the lowest bacterial burden in the lungs and spleen, and those that received P:Δ*caf1* B:rF1-V (0-10% protective) also had significantly lower bacterial burden than the PBS sham-vaccinated group (**Figures 3A, B**). The fraction of mice with bacteremia (**Figure 3C**) reflected the bacterial burden seen in the organs, with all males receiving the P:Δ*caf1* B:rF1-V (0% protective) being bacteremic, in addition to having bacterial burden in the lungs similar to the PBS group.

Among mice given Δ*yopD*/Δ*caf1* vaccine regimens, a similar pattern was seen. Those that received P:rF1-V B:Δ*yopD*/Δ*caf1* (100% protective in females, 90% in males) had significantly lower bacterial burden in the lungs and spleen than the PBS group. Those that received P:Δ*yopD*/Δ*caf1* B:rF1-V (0-20% protective) had significantly lower burden in the spleen, but not in the lungs due to the very high bacterial burden in males, which again were 0% protected by the vaccine (**Figures 3D, E**). All vaccinated females, and 7/8 vaccinated males, had no detectable bacteremia (**Figure 3F**), indicating that even the unsuccessful vaccine regimens enabled some control of systemic bacterial spread from the lungs.

### 2.5 Cytokine levels in lungs of challenged mice compared to sex and bacterial burden

To learn more about the state of infection in male and female mice, we measured the expression of a panel of cytokines in lung and spleen homogenates at 3 dpi. Thirty cytokines were analyzed for lungs, and 27 cytokines for spleens. In this experiment, additional mice were included that had been challenged alongside the mice in **Table 1** (experiments B and C) but received an immunization strategy that protected 0% of both males and females (data not shown). This unsuccessful strategy consisted of one injection of Δ*yopD*/Δ*caf1* or Δ*caf1* LAV, and one injection of approximately 10^8^ radiation-inactivated C12 strain CFU mixed with supernatant containing LcrV protein adsorbed to Alhydrogel. Including these samples, in addition to samples from the vaccine groups shown in **Figures 2**, **3**, increased the sample size to 36 males and 33 females.

Most cytokines had a clear bimodal distribution, with higher levels in samples with greater bacterial burden. For lungs, this bimodal pattern could be captured by categorizing the samples as high-burden (> 10^6^ CFU/g) or low-burden (< 10^3^ CFU/g). For spleens, high-burden was defined as > 8x10^3^ CFU/g and low-burden as < 2x10^3^ CFU/g. **Table 2** shows the levels of all cytokines in lung samples, stratified by sex and bacterial burden (two columns on the left). Interestingly, among lung samples with low bacterial burden, males had a pattern of lower cytokine levels than females, particularly IL-5 and IL-13 (over 5-fold higher in females). For 9/30 cytokines, the average level in females was over twice that in males. This pattern was not seen in lung samples with high bacterial burden.

**Table 2:** Cytokine levels 3 dpi in lung samples from mice challenged with aerosolized *Y. pestis* C12, stratified by sex and by high (> 10^6^ CFU/g) or low (< 10^3^ CFU/g) bacterial burden.

	Females		Males		Females		Males	
	Yp low*	Yp high*	Yp low*	Yp high*	hi/lo ratio	*p*-value **	hi/lo ratio	*p*-value **
**Yp CFU/g**	1.39E+02 (4.39E+01)	6.16E+08 (1.18E+08)	1.22E+02 (5.89E+01)	9.59E+08 (1.18E+08)				
IL-6	28.3 (4.1)	12,559.6 (1,553.7)	23.9 (2.2)	13,136.7 (1,625.2)	443.29	**2.91E-05**	548.77	**4.28E-04**
G-CSF	3.0 (0.6)	262.6 (17.2)	1.8 (0.2)	290.8 (21.0)	88.49	**2.90E-05**	157.80	**4.24E-04**
IFN-γ	11.8 (3.6)	635.9 (87.7)	6.6 (1.6)	598.6 (89.7)	53.67	**2.92E-05**	90.92	**4.28E-04**
IL-1β	12.9 (3.1)	607.6 (64.1)	9.4 (1.8)	551.4 (34.4)	47.20	**2.92E-05**	58.46	**4.28E-04**
IL-1α	17.1 (2.1)	574.7 (102.7)	17.9 (3.1)	359.5 (38.7)	33.58	**2.92E-05**	20.04	**4.28E-04**
LIF	19.0 (4.3)	593.6 (30.4)	8.3 (1.6)	590.9 (23.5)	31.31	**2.92E-05**	71.30	**4.28E-04**
IL-22	43.4 (6.6)	1,118.3 (98.5)	31.3 (6.3)	653.8 (62.9)	25.76	**2.92E-05**	20.91	**4.28E-04**
MIP-1α (CCL3)	18.3 (3.9)	463.1 (68.5)	8.5 (1.1)	499.9 (28.7)	25.36	**2.92E-05**	58.73	**4.28E-04**
GM-CSF	7.1 (1.1)	171.5 (14.1)	4.0 (0.6)	172.7 (15.5)	24.21	**2.92E-05**	42.91	**4.28E-04**
GRO-α (CXCL1)	138.9 (42.2)	3,275.6 (275.6)	58.8 (19.5)	4,213.2 (250.1)	23.59	**2.91E-05**	71.63	**4.21E-04**
TNF-α	4.1 (0.6)	90.4 (8.2)	3.2 (0.3)	94.2 (5.2)	21.80	**2.90E-05**	29.46	**4.27E-04**
IL-17A (CTLA-8)	61.1 (26.3)	664.7 (36.3)	22.9 (8.3)	684.2 (30.9)	10.88	**3.51E-05**	29.81	**4.28E-04**
MIP-1β (CCL4)	36.2 (8.5)	363.4 (50.1)	15.4 (2.8)	509.5 (47.6)	10.04	**2.91E-05**	33.15	**4.28E-04**
M-CSF	1.7 (0.4)	6.9 (0.9)	2.1 (0.7)	6.2 (0.9)	4.06	**1.02E-04**	2.91	**0.022**
IL-27	6.1 (1.1)	20.7 (2.4)	2.5 (0.2)	17.4 (1.4)	3.41	**2.37E-04**	6.88	**4.28E-04**
IL-12p70	6.6 (0.7)	17.4 (1.5)	4.6 (0.7)	14.8 (1.3)	2.64	**3.29E-04**	3.21	**0.002**
ENA-78 (CXCL5)	78.5 (13.1)	182.0 (21.6)	101.1 (25.0)	159.1 (18.3)	2.32	**0.006**	1.57	0.109
IP-10 (CXCL10)	99.2 (17.9)	219.9 (21.3)	60.8 (11.3)	263.6 (16.8)	2.22	**3.29E-04**	4.34	**4.28E-04**
MCP-3 (CCL7)	134.6 (17.6)	283.1 (28.3)	104.3 (19.1)	364.0 (38.7)	2.10	**6.03E-05**	3.49	**4.28E-04**
IL-9	28.6 (3.1)	57.4 (3.3)	25.5 (2.3)	48.9 (2.8)	2.01	**1.02E-04**	1.92	**0.002**
IL-15	9.2 (0.6)	15.6 (1.1)	6.9 (0.9)	13.6 (1.0)	1.68	**0.002**	1.95	**0.004**
IL-10	15.3 (1.3)	24.3 (1.9)	13.3 (1.0)	23.4 (2.4)	1.59	**0.003**	1.75	**0.032**
IL-4	11.9 (1.9)	17.7 (4.3)	4.6 (0.6)	9.7 (1.5)	1.49	0.983	2.11	0.143
IL-2	3.8 (0.3)	5.0 (0.3)	3.6 (0.2)	5.1 (0.5)	1.32	**0.027**	1.40	0.272
RANTES (CCL5)	438.3 (12.7)	415.5 (24.8)	320.8 (34.5)	403.8 (23.6)	0.95	0.413	1.26	0.100
IL-5	59.4 (15.1)	48.9 (9.8)	10.1 (5.2)	38.7 (6.9)	0.82	0.345	3.82	**0.015**
Eotaxin	517.1 (60.9)	399.5 (41.5)	441.7 (92.1)	455.5 (35.9)	0.77	**0.033**	1.03	1.000
IL-23	23.2 (3.8)	16.2 (1.2)	21.3 (2.0)	11.3 (0.6)	0.70	**0.022**	0.53	**7.05E-04**
IL-28	83.8 (11.0)	46.3 (2.9)	78.8 (13.0)	40.5 (0.7)	0.55	**0.002**	0.51	**2.42E-04**
IL-13	36.7 (10.9)	19.7 (1.6)	6.6 (1.8)	15.1 (1.1)	0.54	0.062	2.29	**0.005**
**sample size**	**8**	**25**	**5**	**31**		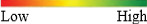		

*All values shown as mean (SEM).

**Mann-Whitney test for comparison of high-burden to low-burden samples within the same sex.

Bolded, *p* < 0.05.

Fold differences between high-burden and low-burden samples is shown in the right-hand columns of **Table 2**. For both males and females, most cytokines were positively correlated with high bacterial burden, many over 10-fold, especially canonical proinflammatory cytokines such as IL-6, IL-1β, and IFN-γ, and others like G-CSF, GM-CSF and GRO-α (CXCL1) that attract and stimulate innate immune cells. A total of 21/30 cytokines were significantly overexpressed (*p* < 0.05) in lungs with high bacterial burden in both males and females. Exceptions to this pattern were ENA-78 (CXCL5) and IL-2 (correlated with bacterial burden in females only); IL-5 and IL-13 (correlated with burden in males only); and RANTES and Eotaxin which had high expression (over 300 pg/mL) in both low- and high-burden samples. The only cytokines significantly lower in organs with high bacterial burden were Eotaxin, IL-28, and IL-23, with this relationship seen for IL-28 and IL-23 in both sexes, and Eotaxin in males only.

Linear regression analyses, using CFU/g to predict the level of each cytokine in the lung tissue, were calculated after natural log (ln) transformation, for the male and female populations. Examples of regression lines are in **Supplementary Figure 2**. The regression coefficients (slope and intercept) were compared between male and female populations to see which cytokines had significant sex differences in the relationship between cytokine and bacterial burden. Slope and intercept, along with sex differences (female minus male) and *p*-values of the sex differences, are shown in **Supplementary Table 3**. For IL-5, IL-13, and GRO-α (CXCL1) the slope and intercept are both significantly different (*p* < 0.05). IL-5 and IL-13 are the only two cytokines where levels decreased with higher bacterial burden in females but increased in males. Also, IP-10 (CXCL10), MIP-1α (CCL3), MIP-1β (CCL4), IL-27, and LIF had *p* < 0.05 between males and females for one of the regression coefficients and *p* < 0.10 for the other.

For comparison across vaccine groups, we removed from analysis the 23 cytokines whose expression in lungs were positively correlated with bacterial burden. Of the remaining 7 cytokines, some trends were of interest, although no comparisons (*n* = 3 or 4) reached statistical significance. In mice vaccinated with P:rF1-V B:Δ*caf1* LAV, females exhibited higher IL-4, IL-5, IL-13 and RANTES levels on average than the corresponding males (**Supplementary Figure 3A**). In mice vaccinated with P:rF1-V B:Δ*yopD*/Δ*caf1* LAV, females had more IL-4, IL-13, IL-2, and Eotaxin, and less ENA-78 (**Supplementary Figure 3B**). In combination with the above data showing that females had less pronounced cytokine overproduction in high-bacterial burden samples compared to low-burden samples, this suggests that females had a better ability to control damaging inflammation in the lungs.

### 2.6 Granulocyte-associated proteins in lungs of challenged mice, compared to sex and bacterial burden

**Figure 4:**
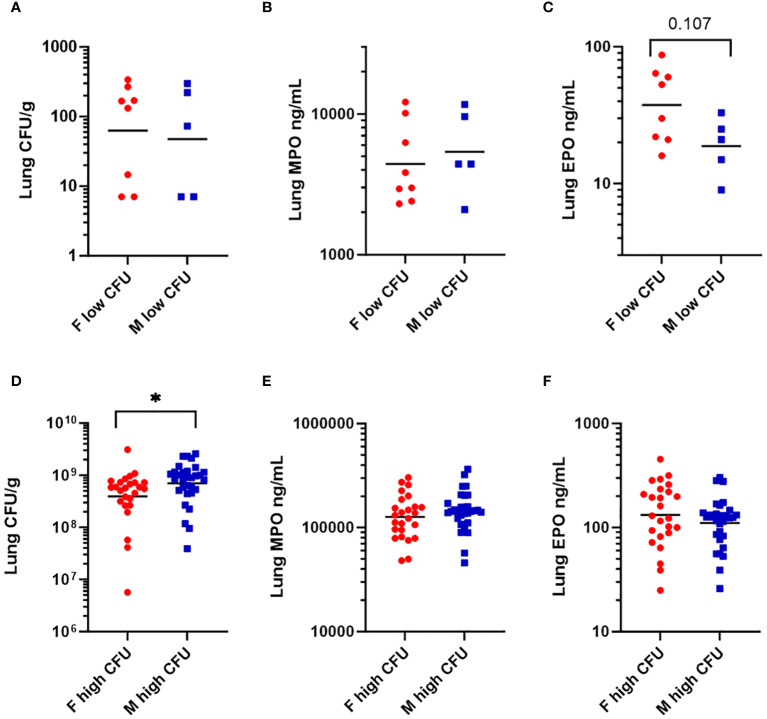
Levels of granulocyte-associated proteins in lung homogenates in mice vaccinated with Δ*caf1* and Δ*yopD*/Δ*caf1* regimens and challenged with aerosolized *Y. pestis* C12. Three dpi, mice were euthanized, and lung homogenates measured for bacteria (CFU/g), myeloperoxidase (MPO ng/mL), and eosinophil peroxidase (EPO ng/mL). Among mice with low bacterial burden (< 10^3^ CFU/g), females (F) and males (M) were compared for bacteria **(A)**, MPO **(B)**, and EPO **(C)**. Among mice with high bacterial burden (> 10^6^ CFU/g), females (F) and males (M) were compared for bacteria **(D)**, MPO **(E)**, and EPO **(F)**. Graphs show each data point and lines representing geometric means. *p < 0.05 in Mann-Whitney test.

Because Th2 cytokines (IL-4, IL-5, IL-13) (52) were higher in females in the low bacterial-burden group than males (**Table 2**), we looked for evidence that females had elevated tissue eosinophils. Additionally, we looked for evidence of elevated tissue neutrophils, having seen that low-burden females also had higher chemokines secreted by neutrophils (GRO-α, IP-10, MIP-1α, MIP-1β) (53). Lung homogenates were quantified for eosinophil peroxidase (EPO) and myeloid peroxidase (MPO), which are released prolifically by eosinophils and neutrophils respectively during antibacterial response (52, 54). Among the low-burden group, females and males had similar bacterial burdens (**Figure 4A**) and MPO levels (**Figure 4B**). EPO was twice as high in females, with a geometric mean of 37.5 compared to 18.8 in males, although the difference was not statistically significant (*p* = 0.107) in a nonparametric test (**Figure 4C**). Among the high-burden group, bacterial burden was significantly higher in males (**Figure 4D**), but males and females had similar MPO and EPO levels (**Figures 4E, F**).

### 2.7 Cytokine levels in spleens of challenged mice, compared to sex and bacterial burden

The levels of all cytokines in spleen samples at 3 dpi were stratified by sex and bacterial burden (**Table 3**, left columns). Unlike in lung samples, the spleen samples with low bacterial burden had similar cytokine levels in males and females, with the only differences greater than 1.5-fold being MCP-3 (CCL7), GRO-α (CXCL1), and G-CSF (higher in males) and MIP-1α (CCL3), MIP-1β (CCL4), IP-10 (CXCL10), and M-CSF (higher in females). This observation was surprising since 10/11 females in the low bacterial burden group (< 2,000 CFU/g) had no detectable bacteria in the spleen, compared to only 4/11 males (**Figures 3B, E**).

**Table 3:** Cytokine levels 3 dpi in spleen samples from mice challenged with aerosolized *Y. pestis* C12, stratified by sex and by high (> 8x10^3^ CFU/g) or low (< 2x10^3^ CFU/g) bacterial burden.

	Females		Males		Females		Males	
	Yp low*	Yp high*	Yp low*	Yp high*	hi/lo ratio	*p*-value **	hi/lo ratio	*p*-value **
**Yp CFU/g**	3.12E+01 (2.42E+01)	1.53E+08 (1.05E+08)	3.14E+02 (1.60E+02)	5.05E+08 (2.61E+08)				
IFN-γ	2.4 (0.2)	838.1 (178.9)	2.2 (0.4)	478.2 (111.8)	350.66	**4.19E-06**	217.50	**7.92E-06**
IL-6	17.3 (5.4)	3,097.2 (1,279.4)	23.3 (9.4)	5,458.6 (1,907.6)	178.52	**3.58E-06**	234.66	**5.53E-06**
GRO-α (CXCL1)	95.6 (46.4)	1,842.4 (369.4)	183.9 (75.3)	2,364.5 (446.6)	19.28	**2.06E-05**	12.86	**5.01E-05**
LIF	3.2 (0.1)	60.6 (18.3)	3.1 (0.0)	92.1 (33.0)	19.12	**4.84E-06**	29.94	**4.13E-05**
IL-1α	8.1 (1.3)	137.8 (33.0)	11.7 (2.1)	148.9 (46.1)	16.95	**4.75E-05**	12.77	**7.83E-05**
IL-22	11.3 (0.4)	183.4 (79.0)	10.9 (0.0)	159.1 (54.5)	16.22	**5.35E-06**	14.61	**5.45E-04**
G-CSF	8.5 (3.6)	127.1 (16.4)	23.1 (9.7)	127.7 (17.5)	14.96	**7.44E-06**	5.53	**8.85E-05**
MCP-3 (CCL7)	19.8 (3.2)	291.3 (58.8)	40.2 (6.8)	256.5 (44.0)	14.74	**4.19E-06**	6.38	**5.81E-06**
IL-1β	8.6 (1.0)	118.0 (34.8)	9.9 (1.5)	113.6 (34.0)	13.79	**3.42E-05**	11.43	**0.001**
IL-17A (CTLA-8)	1.6 (0.0)	10.4 (3.5)	1.6 (0.0)	11.0 (3.5)	6.57	**1.26E-04**	7.00	**1.60E-04**
MIP-1α (CCL3)	36.6 (6.8)	203.2 (37.5)	19.3 (2.7)	188.7 (38.4)	5.55	**0.002**	9.80	**0.009**
IL-27	2.1 (0.0)	11.0 (2.3)	2.0 (0.0)	10.9 (2.6)	5.29	**1.47E-04**	5.34	**1.60E-04**
GM-CSF	3.2 (0.0)	14.4 (2.8)	3.1 (0.0)	13.4 (3.9)	4.55	**0.002**	4.27	**0.002**
TNF-α	2.8 (0.0)	12.1 (3.3)	2.8 (0.0)	14.5 (4.2)	4.35	**0.011**	5.21	**0.006**
IP-10 (CXCL10)	59.8 (6.5)	240.1 (27.1)	38.9 (5.9)	204.4 (25.4)	4.01	**3.42E-05**	5.25	**2.08E-04**
Eotaxin	135.7 (7.4)	433.9 (55.9)	150.7 (13.8)	363.0 (50.1)	3.20	**3.56E-05**	2.41	**6.45E-04**
IL-4	2.1 (0.2)	5.6 (1.9)	1.4 (0.1)	5.5 (1.4)	2.68	0.260	3.83	**0.047**
IL-13	3.1 (0.0)	6.9 (1.1)	3.1 (0.0)	5.6 (0.8)	2.25	**0.002**	1.83	**0.027**
MIP-1β (CCL4)	103.0 (17.4)	219.2 (52.0)	63.0 (7.6)	189.7 (48.4)	2.13	0.188	3.01	0.192
IL-12p70	2.0 (0.1)	4.0 (0.7)	1.9 (0.0)	3.6 (0.5)	1.99	**0.012**	1.87	**0.008**
IL-10	4.4 (0.4)	7.7 (0.7)	3.7 (0.3)	7.3 (0.7)	1.76	**0.005**	1.99	**0.002**
IL-15	2.3 (0.0)	3.9 (0.4)	2.3 (0.0)	3.6 (0.5)	1.70	**0.002**	1.61	**0.007**
IL-2	1.6 (0.0)	2.7 (0.3)	1.6 (0.0)	2.2 (0.2)	1.69	**0.001**	1.37	**0.010**
IL-5	2.5 (0.0)	4.0 (0.5)	2.5 (0.0)	3.2 (0.3)	1.59	**0.004**	1.26	0.059
M-CSF	3.3 (0.6)	3.7 (0.8)	1.4 (0.4)	2.1 (0.5)	1.13	0.985	1.46	0.915
ENA-78 (CXCL5)	80.4 (9.0)	77.9 (8.0)	65.7 (9.3)	105.2 (7.8)	0.97	0.804	1.60	**0.005**
RANTES (CCL5)	533.5 (14.6)	442.3 (17.0)	437.3 (30.0)	427.0 (12.5)	0.83	**2.65E-04**	0.98	0.149
**sample size**	**11**	**22**	**11**	**25**		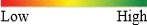		

*All values shown as mean (SEM).

**Mann-Whitney test for comparison of high-burden to low-burden samples within the same sex. Bolded, *p* < 0.05.

Fold differences between high-burden and low-burden samples are shown in the right-hand columns of **Table 3**. Even more so than in lungs, cytokines were overexpressed in spleens with high bacterial burdens, with this overexpression being significant (*p* < 0.05) for both sexes in 21 of 27 cytokines.

Linear regressions of ln-transformed data were also performed for cytokine levels in spleen samples. In comparing male and female regression coefficients (**Supplementary Table 4**), females had higher intercepts for most cytokines, with several being significantly higher. The only cytokines with a significantly different slope of the regression line were ENA-78 (CXCL5) and IP-10 (CXCL10), where overexpression in high-burden samples was more pronounced in males. These results differ from the cytokine results in lung samples. In lungs, there was a pattern of females having higher cytokine levels at low bacterial burden but males having more upregulation at high bacterial burden, while in spleens both sexes had similar upregulation from low to high.

### 2.8 Humoral immunity elicited by vaccines

#### 2.8.1 Female mice given rF1-V prime and LAV boost have enhanced serum antibody titers against rF1-V and rV

Approximately four weeks post-boost, four mice from each group were euthanized pre-challenge and serum was isolated from blood taken from axillary vessels. Levels of total IgG, IgG1, and IgG2a were measured against *Y. pestis* antigens rF1-V, rV, or irradiated whole *Y. pestis* cells of temperature-shifted CO92 (TS CO92) or C12 (TS C12) strains. All vaccinated groups had significantly more serum IgG against rF1-V and rV than did sham-vaccinated mice. Furthermore, females had more IgG against rF1-V (**Table 4**) and more IgG against rV (**Table 5**) than corresponding males for three of four vaccine conditions, although the sex differences were not all significant. Regarding IgG2a and IgG1 serology, which reflect Th1 and Th2 immunity respectively, the vaccine regimens with rF1-V as prime had higher IgG2a/IgG1 ratios than those with rF1-V as boost, suggesting a more balanced Th1/Th2 response to the latter vaccine regimen compared to the Th2-polarized response to the former regimen (**Tables 4**, **5**).

**Table 4:** Serum antibody titers against *Y. pestis* rF1-V antigen in mice vaccinated with Δ*caf1* or Δ*yopD*/Δ*caf1* regimens.

Sex	Vaccine Prime	Vaccine Boost	21-day Survival	*n* =	Anti-rF1-V Antibody Titer^a^			
						Isotype		Ratio IgG2a/IgG1
					IgG	IgG1	IgG2a	
Female	PBS	PBS	0%	4	84 (1.60)	100 (1.48)	50 (1.00)	n/a
Male	PBS	PBS	0%	4	50 (1.00)	89 (1.59)	50 (1.00)	n/a
Female	rF1-V	Δ*caf1*	100%	4	**1,208,159 (1.32)**	**14,367,514 (1.22)**	**678,056 (1.26)**	0.047
Male	rF1-V	Δ*caf1*	50%	4	**134,543 (1.40)**	**1,076,347 (1.52)**	**4,525 (1.37)**	0.004
Female	Δ*caf1*	rF1-V	10%	3	**592,560 (1.60)**	4,377,538 (2.19)	32,084 (4.59)	0.007
Male	Δ*caf1*	rF1-V	0%	4	**169,514 (1.20)**	905,097 (1.20)	3,200 (1.39)	0.004
Female	PBS	PBS	0%	4	50 (1.00)	50 (1.00)	50 (1.00)	n/a
Male	PBS	PBS	0%	4	50 (1.00)	50 (1.00)	50 (1.00)	n/a
Female	rF1-V	Δ*yopD*/Δ*caf1*	100%	4	*403,175 (1.25)*	*403,175 (1.49)*	*57,243 (1.94)*	0.142
Male	rF1-V	Δ*yopD*/Δ*caf1*	90%	4	359,188 (1.34)	854,398 (1.58)	80,635 (2.00)	0.094
Female	Δ*yopD*/Δ*caf1*	rF1-V	20%	4	*45,255 (1.33)*	*42,546 (1.45)*	*424 (1.85)*	0.010
Male	Δ*yopD*/Δ*caf1*	rF1-V	0%	4	142,544 (1.90)	142,544 (1.55)	4,776 (2.66)	0.034

a Values represent geometric mean with geometric standard error in parentheses.

Bolded, *p* < 0.05 in Mann-Whitney comparison of males to females with same regimen. Italicized, *p* < 0.05 in Mann-Whitney comparison of (rF1-V prime: LAV boost) to (LAV prime: rF1-V boost) among same sex.

**Table 5:** Serum antibody titers against *Y. pestis* rV antigen in mice vaccinated with Δ*caf1* or Δ*yopD*/Δ*caf1* regimens.

Sex	Vaccine Prime	Vaccine Boost	21-day Survival	*n* =	Anti-rV Antibody Titer^a^			
						Isotype		Ratio IgG2a/IgG1
					IgG	IgG1	IgG2a	
Female	PBS	PBS	0%	4	84 (1.60)	178 (2.21)	50 (1.00)	n/a
Male	PBS	PBS	0%	4	59 (1.17)	50 (1.00)	50 (1.00)	n/a
Female	rF1-V	Δ*caf1*	100%	4	**574,701 (1.28)**	**4,872,974 (1.30)**	**48,326 (1.26)**	0.010
Male	rF1-V	Δ*caf1*	50%	4	**80,635 (1.53)**	**430,539 (1.64)**	**2,016 (1.78)**	0.005
Female	Δ*caf1*	rF1-V	10%	3	322,540 (2.39)	3,002,095 (1.94)	5,486 (3.59)	0.002
Male	Δ*caf1*	rF1-V	0%	4	161,270 (1.25)	542,445 (1.32)	3,200 (1.34)	0.006
Female	PBS	PBS	0%	4	50 (1.00)	50 (1.00)	50 (1.00)	n/a
Male	PBS	PBS	0%	4	50 (1.00)	50 (1.00)	50 (1.00)	n/a
Female	rF1-V	Δ*yopD*/Δ*caf1*	100%	4	683,605 (2.01)	*2,581,158 (1.36)*	*12,800 (3.20)*	0.005
Male	rF1-V	Δ*yopD*/Δ*caf1*	90%	4	514,028 (1.43)	2,169,780 (1.54)	38,205 (2.72)	0.018
Female	Δ*yopD*/Δ*caf1*	rF1-V	20%	4	53,817 (1.48)	*341,719 (1.37)*	*100 (1.44)*	0.0003
Male	Δ*yopD*/Δ*caf1*	rF1-V	0%	4	144,815 (1.76)	512,000 (1.67)	1,068 (3.10)	0.002

a Values represent geometric mean with geometric standard error in parentheses.

Bolded, *p* < 0.05 in Mann-Whitney comparison of males to females with same regimen. Italicized, *p* < 0.05 in Mann-Whitney comparison of (rF1-V prime: LAV boost) to (LAV prime: rF1-V boost) among same sex.

When antibody titers against TS CO92 (encapsulated) and TS C12 (nonencapsulated) irradiated *Y. pestis* were measured, the pattern of female mice having higher titers was not seen, nor was the pattern of mice given rF1-V prime having higher IgG2a/IgG1 ratios (**Table 6**). Interestingly, titers against these whole-cell *Y. pestis* antigens were notably lower in mice given Δ*yopD*/Δ*caf1* LAV than in the corresponding groups vaccinated with Δ*caf1* LAV. We had hypothesized that deleting *yopD* could enhance production of V antigen, which is negatively regulated by YopD, while reducing YopD-driven secretion of immunosuppressive factors, making the strain more antigenic (46, 55); however, this idea was not supported by the serology data.

**Table 6:** Serum antibody titers against irradiated whole-cell *Y. pestis* TS CO92 and TS C12 antigens in mice vaccinated with Δ*caf1* or Δ*yopD*/Δ*caf1* regimens.

Sex	Vaccine Prime	Vaccine Boost	21-day Survival	*n* =	Anti-TS CO92 Antibody Titer^a^	Anti-TS C12 Antibody Titer^a^			
							Isotype		Ratio IgG2a/IgG1
					IgG	IgG	IgG1	IgG2a	
Female	PBS	PBS	0%	4	50 (1.00)	50 (1.00)	56 (1.14)	50 (1.00)	n/a
Male	PBS	PBS	0%	4	59 (1.17)	71 (1.37)	50 (1.00)	50 (1.00)	n/a
Female	rF1-V	Δ*caf1*	100%	4	*30,444 (1.30)*	2,851 (1.99)	9,589 (2.20)	63 (1.25)	0.007
Male	rF1-V	Δ*caf1*	50%	4	19,178 (1.20)	2,691 (1.36)	6,375 (3.37)	424 (2.53)	0.066
Female	Δ*caf1*	rF1-V	10%	3	*87,320 (1.47)*	34,653 (1.19)	172,810 (1.27)	2,177 (1.23)	0.013
Male	Δ*caf1*	rF1-V	0%	4	30,264 (1.52)	7,611 (2.46)	21,442 (2.20)	356 (2.39)	0.017
Female	PBS	PBS	0%	4	50 (1.00)	50 (1.00)	50 (1.00)	50 (1.00)	n/a
Male	PBS	PBS	0%	4	50 (1.00)	50 (1.00)	50 (1.00)	50 (1.00)	n/a
Female	rF1-V	Δ*yopD*/Δ*caf1*	100%	4	4,525 (1.37)	100 (1.56)	126 (2.17)	50 (1.00)	0.397
Male	rF1-V	Δ*yopD*/Δ*caf1*	90%	4	5,382 (1.48)	168 (2.04)	267 (2.53)	71 (1.37)	0.266
Female	Δ*yopD*/Δ*caf1*	rF1-V	20%	4	3,390 (1.54)	100 (1.87)	200 (2.87)	50 (1.00)	0.250
Male	Δ*yopD*/Δ*caf1*	rF1-V	0%	4	1,796 (1.73)	119 (1.81)	150 (1.89)	50 (1.00)	0.333

a Values represent geometric mean with geometric standard error in parentheses.

Italicized, *p* < 0.05 in Mann-Whitney comparison of (rF1-V prime: LAV boost) to (LAV prime: rF1-V boost) among same sex.

#### 2.8.2 After aerosol challenge, tissue bacterial burden correlates with having low serum antibody titers against rF1-V and rV

**Figure 5:**
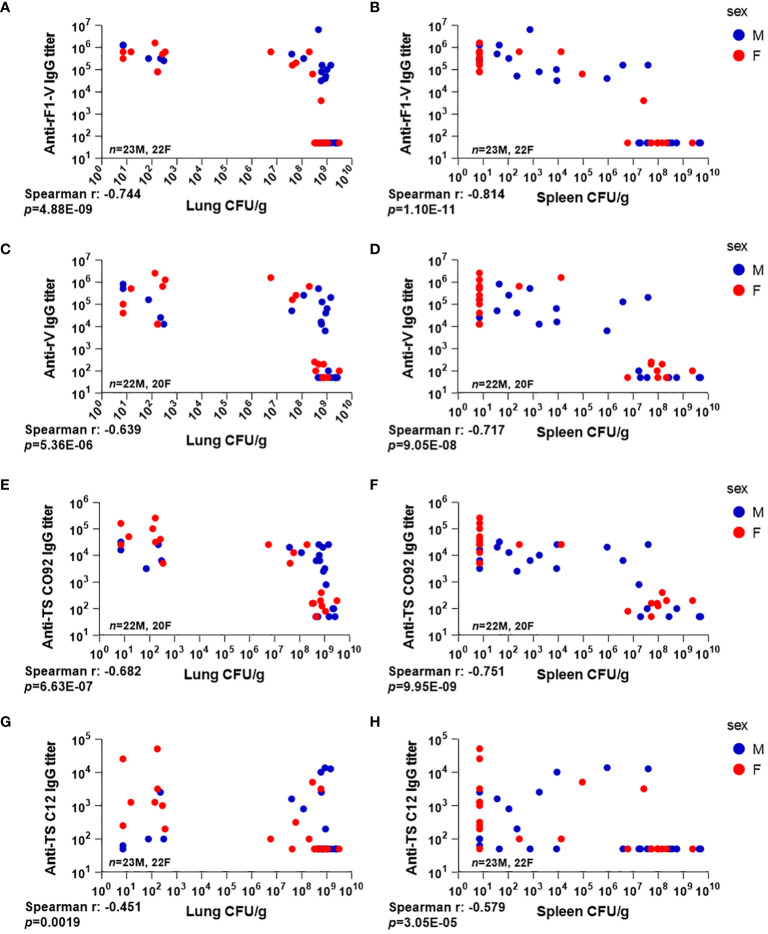
Serum antibody levels compared to tissue bacterial burden in mice vaccinated with Δ*caf1* and Δ*yopD*/Δ*caf1* regimens and challenged with aerosolized *Y. pestis* C12. Three dpi, male (M) and female (F) mice were euthanized; lung and spleen homogenates were plated to quantify bacteria (CFU/g); and serum samples were measured for total IgG titer against rF1-V **(A, B)**, rV **(C, D)**, and temperature-shifted whole-cell CO92 **(E, F)** and C12 **(G, H)** antigens. Serum IgG titer for each mouse was plotted against bacterial burden in lungs **(A, C, E, G)** and spleens **(B, D, F, H)**.

Total IgG against the above antigens was measured 3 dpi in the same mice that were euthanized for bacterial quantitation (*n* = 45). Detectable anti-rF1-V IgG antibody titers were observed in all mice that received any heterologous vaccine regimen (29/45), while all of those that received PBS (16/45) had baseline or undetectable titers along with high bacterial burden in lungs. Among those with detectable anti-rF1-V titer, 13/29 had low bacterial burden in lungs, and 16/29 had a detectable antibody titer but also a bacterial burden over 10^6^ CFU/g (**Figure 5A**). Of note, all the male mice that received P:Δ*caf1* B:rF1-V or P:Δ*yopD*/Δ*caf1* B:rF1-V, vaccine strategies that failed to protect any males, still had high titers against rF1-V, also seen in pre-challenge titers (**Table 4**), and yet we observed uncontrolled bacterial replication in lungs (**Figures 3A, D**).

A similar pattern was seen in spleens (**Figure 5B**), although many of the mice with uncontrolled bacterial replication in lungs had limited bacterial replication in the spleen (compare the clusters of datapoints in the top right of **Figures 5A, B**). The same pattern was seen with antibody titers to rV antigen alone (**Figures 5C, D**), and to TS CO92 (**Figures 5E, F**). However, antibody titers against TS C12 did not follow this pattern, with several mice having low bacterial burden despite low or undetectable anti-TS C12 titers (**Figures 5G, H**). These data further support the utility of anti-rF1-V antibodies in fighting aerosolized *Y. pestis*, even with an F1-negative challenge strain.

#### 2.8.3 Complement activation in males and females during aerosol challenge

Because sex differences in complement activation have been consistently observed in mice and humans (56, 57), we examined that aspect of innate immunity. We measured serum levels of C3a, a pro-inflammatory anaphylatoxin, and C5b-9, the pore-forming complex that represents terminal complement activation and has bactericidal effect, with elevated soluble C5b-9 complexes in circulation being associated with bacterial infection. Both factors were positively correlated to bacterial burden in the tissues (**Supplementary Figure 4A-B, D-E**). Both vaccinated and PBS-treated males had higher levels of C3a and C5b-9 relative to female mice. In addition, the levels of both C3a and C5b-9 were reduced in those vaccinated, relative to PBS counterparts, with a greater percent reduction observed in the females (C3a 62% female vs. 49% male, and C5b-9 51% female vs. 41% male) (**Supplementary Figure 4C, 4F**).

### 2.9 Cellular immunity elicited by vaccines

#### 2.9.1 Lymphocyte activation by *ex vivo* antigen stimulation of splenocytes

**Figure 6:**
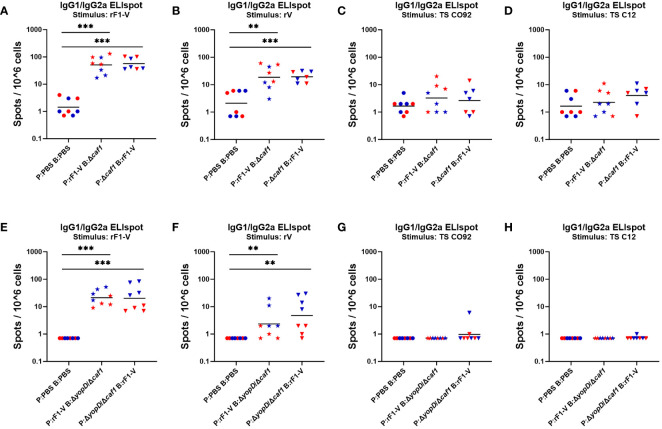
ELISpot assays for IgG-secreting splenocytes from mice given Δ*caf1* and Δ*yopD*/Δ*caf1* vaccine regimens, after ex vivo re-stimulation with *Y. pestis* antigens. Four weeks post-boost, female (red) and male (blue) mice were euthanized and splenocytes re-stimulated with B-Poly S Polyclonal B cell Stimulator for 5 days, then 8 h with rF1-V **(A, E)**, rV **(B, F)**, or temperature-shifted whole-cell CO92 **(C, G)** and C12 **(D, H)** antigens, followed by quantification of spots representing cells secreting soluble IgG1 and IgG2a. Splenocytes from mice given Δ*caf1* vaccine regimens are shown in **(A–D)**; splenocytes from mice given Δ*yopD*/Δ*caf1* vaccine regimens are shown in E-H. Graphs show each data point and lines representing geometric means. **p < 0.01, ***p < 0.001 in Mann-Whitney test.

Four weeks post-boost, mice (*n* = 4) from each group were euthanized pre-challenge, spleens were taken and splenocytes isolated through gentle homogenization. ELISpot assays were performed to quantify B cells secreting soluble IgG1 and IgG2a in response to *Y. pestis* antigens rF1-V, rV, TS CO92 and TS C12. Among mice given any Δ*caf1* or Δ*yopD*/Δ*caf1* vaccine regimen, the number of IgG-secreting splenocytes upon rF1-V stimulation was significantly higher than in sham-vaccinated mice (**Figures 6A, E**). This pattern was also seen in splenocytes stimulated with rV (**Figures 6B, F**). In those given Δ*yopD*/Δ*caf1* vaccine regimens, males surprisingly had more IgG-secreting splenocytes than corresponding females (**Figures 6E, F**), but this was not seen in those given Δ*caf1* vaccine regimens (**Figures 6A, B**). If we compare Δ*caf1*-immunized mice (**Figures 6A, B**) to Δ*yopD*/Δ*caf1*-immunized mice (**Figures 6E, F**), one interpretation could be that males had similar numbers of IgG-secreting splenocytes after all vaccine regimens, while females immunized with Δ*caf1* had more IgG-secreting splenocytes than those given Δ*yopD*/Δ*caf1*. Unlike with rF1-V or rV stimulation, the number of IgG-secreting cells after stimulation with TS CO92 or TS C12 was close to background in all cases (**Figures 6C, D, G, H**).

Additional ELISpot assays were performed to quantify splenocytes that produced IFN-γ in response to the same antigens as above (**Supplementary Figure 5**). Among mice given any Δ*caf1* or Δ*yopD*/Δ*caf1* vaccine regimen, all vaccinated groups had more IFN-γ-secreting splenocytes than did sham-vaccinated mice when stimulated by rF1-V (**Figures S5A**, **S5E**) or rV (**Figures S5B**, **S5F**), although the significance was not as strong as that seen for IgG-secreting splenocytes.

When cells were stimulated by TS CO92 (**Figures S5C**, **S5G**) or TS C12 (**Figures S5D**, **S5H**), there were detectable IFN-γ-secreting splenocytes, unlike IgG-secreting splenocytes, but most vaccinated groups still had geometric means that were not above the levels seen in sham-vaccinated mice. In general, the data for IFN-γ-secreting splenocytes (“T cell ELISpot”) were less conclusive than the data for IgG-secreting splenocytes (“B cell ELISpot”), and B cell ELISpot results correlated with serology and vaccine protection while T cell ELISpot results did not. This suggests that protection conferred by the vaccine is more correlated with humoral immunity than with T cell immunity, at least at the timepoints we analyzed in this study.

#### 2.9.2 Cytokines induced by *ex vivo* antigen stimulation of splenocytes

Approximately four weeks post-boost, mice (*n* = 4) from each group were euthanized pre-challenge, spleens were taken and splenocytes isolated through gentle disassociation. After red blood cell lysis, equal numbers of cells from each spleen were stimulated for 44-48 h with antigen or medium only. The antigen used was rF1-V or TS C12. For this analysis, females and males were considered separately.

When splenocytes were stimulated with rF1-V, all immunization groups showed an increase for most cytokines, above the levels elicited in mice given sham PBS immunizations (**Supplementary Tables 5, 6**). Among mice given Δ*caf1* vaccine regimens (**Table S5**), the females given P:rF1-V B:Δ*caf1* (100% protective), were distinguished by having less elevated cytokine expression than those given the 10% protective P:Δ*caf1* B:rF1-V regimen. However, this trend was not seen with males. Male mice given P:rF1-V B:Δ*caf1* (50% protective) had higher expression on average than males given the non-protective P:Δ*caf1* B:rF1-V regimen.

Among mice given Δ*yopD*/Δ*caf1* vaccine regimens (**Table S6**), there were fewer differences in cytokine induction between the four vaccine groups. However, the same pattern was seen, in which females given P:rF1-V B:Δ*yopD*/Δ*caf1* (100% protective) had lower induction of most cytokines compared to the females given P:Δ*yopD*/Δ*caf1* B:rF1-V (10% protective). Also, males given P:rF1-V B:Δ*yopD*/Δ*caf1* (90% protective) had higher expression of most cytokines than males given the non-protective P:Δ*yopD*/Δ*caf1* B:rF1-V regimen. **Supplementary Table 7** shows these data expressed as ratio of vaccine group to PBS group, to factor in the background level of cytokine secretion by splenocytes from sham-vaccinated mice.

When splenocytes were stimulated with irradiated TS C12 *Y. pestis*, the observed cytokine levels were higher than with rF1-V stimulus, but the increase in vaccine groups above the PBS group was less, due to nonspecific stimulation in all groups including the PBS group (**Supplementary Tables 8, 9**). However, several cytokines were still expressed at levels above the PBS group, which can be seen when the data are presented as ratio of vaccine group to PBS group (**Supplementary Table 10**). The three most overexpressed cytokines in vaccine groups were IL-17A, IL-2, and IL-4, for which the overexpression was less pronounced in females receiving 100% protective vaccines (P:rF1-V B:Δ*caf1* or P:rF1-V B:Δ*yopD*/Δ*caf1*). In these data, one repeated pattern is that females given the most protective vaccine regimens had less cytokine induction upon splenocyte restimulation than females given the vaccine doses in the reverse order that was not protective – and this pattern was not seen for males.

#### 2.9.3 CD44 upregulation in splenic T cells

Approximately four weeks post-boost, groups of mice (*n* = 4) from each group were euthanized pre-challenge and splenocytes were cryopreserved for phenotyping by flow cytometry. Data were analyzed using the following gating strategy (example shown in **Supplementary Figure 6**). Splenic T cells were identified as CD3+ CD19- cells, and then categorized as either CD4+ or CD8+. CD4+ or CD8+ T cells were then categorized for surface expression of CD44, a widely expressed glycoprotein associated with T cell-APC interactions, tissue homing, activation, and memory T cell phenotype (58, 59). We saw a consistent pattern of female mice having a higher percentage of CD44+ T cells, among both the CD8+ and CD4+ T cell populations (**Table 7**). This was visible in all groups, including PBS sham vaccination. Among female mice, the percentage of CD44+ T cells was elevated above sham in mice receiving P:rF1-V B:Δ*yopD*/Δ*caf1* and P:Δ*yopD*/Δ*caf1* B:rF1-V, but this was not seen with the mice receiving Δ*caf1* (**Table 7**).

**Table 7:** CD44 surface expression on CD4+ and CD8+ T cells from spleens of mice immunized with Δ*caf1* or Δ*yopD*/Δ*caf1* vaccine regimens.

Sex	Vaccine Prime	Vaccine Boost	*n* =	CD4+ CD44+ T cells (%)*	CD8+ CD44+ T cells (%)*
				Mean (SEM)	Mean (SEM)
Female	PBS	PBS	4	**21.4 (1.5)**	14.7 (0.8)
Male	PBS	PBS	4	**16.1 (1.0)**	12.5 (1.2)
Female	rF1-V	Δ*caf1*	4	18.2 (0.9)	14.0 (1.7)
Male	rF1-V	Δ*caf1*	4	17.0 (0.7)	10.6 (0.8)
Female	Δ*caf1*	rF1-V	3	22.4 (1.2)	*17.4 (1.4)*
Male	Δ*caf1*	rF1-V	4	21.4 (2.1)	*8.8 (0.7)*
Female	PBS	PBS	4	16.2 (1.2)	11.0 (1.1)
Male	PBS	PBS	4	13.4 (1.7)	8.1 (1.3)
Female	rF1-V	Δ*yopD*/Δ*caf1*	4	*18.0 (1.8)*	**14.2 (1.1)**
Male	rF1-V	Δ*yopD*/Δ*caf1*	4	*13.7 (0.7)*	**7.9 (0.7)**
Female	Δ*yopD*/Δ*caf1*	rF1-V	4	19.5 (3.8)	13.4 (1.0)
Male	Δ*yopD*/Δ*caf1*	rF1-V	4	15.3 (0.5)	12.4 (2.1)

*Number of CD44+ cells as fraction of total CD4+ or CD8+ T cells. Bolded, *p* < 0.05 in Mann-Whitney comparison of males and females. Italicized, *p* < 0.07.

In another splenocyte phenotyping panel, dendritic cells (DCs) were identified as CD3-, CD19-, and CD11c+ cells, and then categorized as either CD8+ or CD11b+, with CD8+ more associated with Th1 immunity and cross-presentation to CD8+ T cells, and CD11b+ more associated with Th2 immunity and MHC II presentation, among other phenotypes (60, 61). DC populations were categorized for their surface expression of activation markers CD40, CD80 and CD86. No consistent pattern could be seen that distinguished those that received protective vaccine regimens from others or distinguished males from females (data not shown).

One factor complicating the flow cytometry data was that live/dead cell staining could not be used, reducing the signal/noise ratio by including dead cells in populations. This also interfered with staining of CD62L, which in our hands is a more reliable marker to distinguish naïve (CD62L+ CD44-) from memory (CD62L- CD44+) T cells but could not be used in this analysis to support the CD44 staining data.

### 2.10 Layered defense strategies can rescue female, but not male, BALB/c mice immunized with suboptimal vaccination strategies

**Figure 7:**
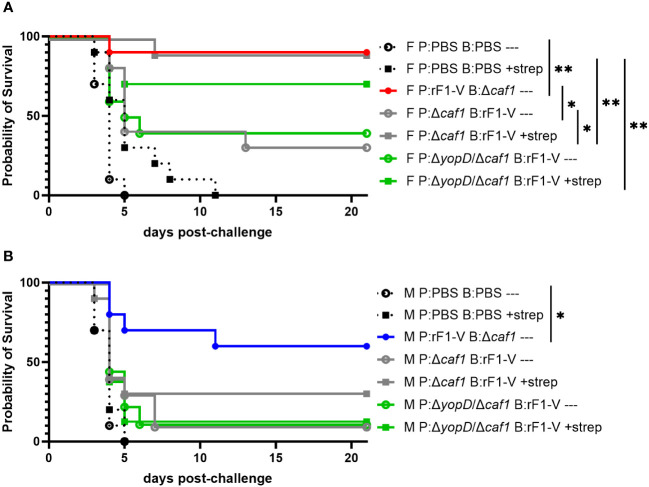
Protection of BALB/c mice against lethal challenge with aerosolized *Y. pestis* C12 by a vaccination and post-challenge antibiotic treatment strategy. **(A)** Female mice (n=10/group) were immunized with PBS only; P:rF1-V B: Δ*caf1* (optimal regimen); or regimens with P: Δ*caf1* or P: Δ*yopD*/Δ*caf1* and B: rF1-V (suboptimal regimens). All mice were challenged with C12 (6.7 x 10^5^ CFU) at 31 d post-boost. Mice immunized with PBS or suboptimal regimens were given attempted rescue doses of streptomycin (20 mg/kg every 6 h) starting 60 h post-boost. **(B)** Male mice (*n*=8-10/group) were immunized, challenged with C12 (7.7 x 10^5^ CFU), and given streptomycin as described in **(A)** Clinical progression was monitored daily for 21 days. *p < 0.05, **p < 0.01 in pairwise comparison of groups by probability of survival (%) using a Fisher exact test.

We have previously demonstrated the power of layering medical countermeasures in a pneumonic plague model (34). In those studies, we demonstrated that suboptimal vaccination regimens combined with suboptimal (delayed) antibiotic treatments can lead to statistically significant synergy and a dramatic increase in survival rates over vaccine or antibiotic alone. Here we sought to corroborate our previous work by vaccinating with the less protective regimens used in the current studies, and then administering streptomycin to see if this could augment the suboptimal level of protection observed. In this experiment we gave mice suboptimal vaccine regimes, challenged them with aerosolized *Y. pestis* C12 four weeks post-boost, and then attempted to rescue the challenged mice with streptomycin treatment starting 60 h post-challenge (**Table 1**, experiment D). The positive-control vaccination sequence (P:rF1-V B:Δ*caf1*), even without streptomycin, conferred 90% protection in female mice and 60% protection in male mice. Suboptimal vaccine regimes alone (P:Δ*caf1* B:rF1-V or P:Δ*yopD*/Δ*caf1* B:rF1-V) protected 30%-40% of female mice, and streptomycin alone only delayed the time to mortality. However, when layered together these suboptimal countermeasures protected 70%-90% of the female mice, significantly more than streptomycin alone (**Figure 7A**). The layering of medical countermeasures resulted in statistically significant synergy in female mice immunized with P:Δ*caf1* B:rF1-V (*p* = 0.0017) or nearly significant synergy in female mice immunized with P:Δ*yopD*/Δ*caf1* B:rF1-V (*p* = 0.0575), using the Bliss independence model to calculate synergy scores (62). However, in males, when streptomycin was given to mice immunized with a suboptimal vaccine, still only 12%-30% of males were protected. In males the layering of suboptimal vaccine and streptomycin provided no significant benefit beyond streptomycin or vaccine alone, with no evidence of Bliss synergy (**Figure 7B**).

## 3 Discussion

### 3.1 Discussion of results

*Y. pestis* is a re-emerging threat due to the existence of natural plague foci in many countries, its potential as a biothreat agent, rapid progression to severe disease, and acquisition of antibiotic resistance. Furthermore, although most wild-type strains produce a robust outer capsule composed of polymeric F1, natural isolates deficient in capsule production have been identified (63–65) and studies confirmed that capsule production is not essential for virulence (22, 34, 66, 67). The most established vaccine platforms rely on anti-F1 and anti-LcrV immune responses to control infection. An immune response against F1 will not protect against capsule-deficient strains and thus, protection against these strains would only be attributed to anti-LcrV response. There is a great need for effective medical countermeasures that include novel vaccine formulations and schedules that provide robust protection against both capsule-producing and capsule-deficient strains.

To this end we developed two novel LAVs (*pgm-* pPst- Δ*caf1* and *pgm-* pPst- Δ*yopD*/Δ*caf1*) with the aim of enriching for protective antigens exclusive of the F1 protein. Importantly, these results provide experimental data in mice that can be used to develop or refine next-generation vaccination strategies. We acknowledge the inherent difficulty in manufacturing and delivering heterologous vaccines, including but not limited to: requirement of separate manufacturing streams, the disparate requirements for ensuring quality control to produce protein subunit and LAV vaccines, and even separate distribution and delivery requirements. While heterologous vaccination strategies are clearly more complex than traditional homologous approaches, this work highlights their potential utility to combat isolates of bacteria with varying virulence attributes, which is of particular importance in context of antimicrobial resistance, engineered threats, and potential emerging or re-emerging bacterial pathogens.

A second objective of this work was to corroborate and further characterize the sex differences in vaccine protective efficacy after intranasal challenge with *Y. pestis* reported by Bowen et al. (47) in both BALB/c and C57BL/6 mice. These differences were an important, albeit hard to explain, observation and clearly must be considered when using a mouse model to evaluate medical countermeasures against a biodefense pathogen.

We tested the novel LAVs using heterologous vaccination strategies combining a dose of LAV with a dose of rF1-V subunit vaccine, 3-4 weeks apart. We saw that the order of administration of the two doses was critical: priming with rF1-V subunit, followed by either Δ*caf1* or Δ*yopD*/Δ*caf1* LAV boost, was more protective than LAV prime followed by rF1-V boost for both males and females. Priming with the subunit vaccine offers clear advantage in this model of pneumonic plague with the vaccines described in this study. This vaccine strategy may provide a more robust stimulation of memory B cells with a more effective booster immune response due to the LAV producing native rather than recombinant proteins. Priming with either LAV and boosting with rF1-V led to no more than 20% protection, differing from our recent study where priming with Combo Δ*yscN* LAV and boosting with rF1-V conferred 90% protection in female mice (34). One explanation could be that the Δ*yscN* strains express a wider array of *Y. pestis* proteins due to their retention of the *pgm* locus and pPst plasmid (11, 48). In contrast, Δ*caf1* and Δ*yopD*/Δ*caf1* are on a *pgm-* pPst- background. The Δ*yscN* strains do have greatly impaired virulence (37–39) as they are unable to secrete Yops through the T3SS. This sequestration of the Yops might also assist the development of immune memory because of the anti-inflammatory, anti-phagocytic and otherwise immune suppressive functions of Yops (68).

This study builds on other studies on heterologous prime-boost immunization, against *Y. pestis* (69) or other pathogens (70–73), showing major differences in immunity depending on which formulation is administered first. Our studies found that female mice were more protected than males by these heterologous vaccines, whether they included *pgm-* pPst- Δ*yopD*/Δ*caf1*, *pgm-* pPst- Δ*caf1*, or CO92 Δ*yscN* + C12 Δ*yscN* as the LAV. Especially interesting was that in addition to enhanced vaccine protection, female mice were more responsive to a post-challenge antibiotic treatment designed to be ineffective for sham-vaccinated mice, but rescue those with partial vaccine protection (**Figure 7**). These data demonstrate that the disadvantage observed in the vaccinated male mice is substantial enough that it cannot be augmented with antibiotic treatment in the same manner as females.

We saw more male survival with the Δ*yopD*/Δ*caf1* vaccine (90%) than the Δ*caf1* vaccine (50%). However, the Δ*caf1* vaccine experiment used more than double the challenge dose, so we would not consider this evidence that the Δ*yopD*/Δ*caf1* vaccine is more effective, especially given the serology and ELISpot data. Difference in challenge doses may also account for the slight increase in bacterial dissemination from the lungs, as seen with more Δ*caf1* vaccinated mice having bacteremia than corresponding Δ*yopD*/Δ*caf1* vaccinated mice.

Bacterial burden in lungs was low (compared to sham immunization) in both males and females receiving rF1-V prime/LAV boost vaccines, and was similarly low in the females receiving LAV prime/rF1-V boost vaccines that were only 10-20% protective. Males receiving LAV prime/rF1-V boost vaccines, however, had high bacterial burden in lungs, like sham-vaccinated mice. However, even these males had less bacteria in the spleen than sham-vaccinated mice. This could be due to vaccine-associated mitigation or delay of systemic dissemination of bacteria at 3 dpi, without controlling bacterial replication in the lungs. Supporting this, for the mice where bacterial replication in the lungs was uncontrolled despite having high anti-rF1-V antibody titers (**Figure 6A**), bacteria in the spleen were partially controlled (**Figure 6B**).

When tissue cytokine levels were measured in C12-challenged mice, we saw that most of the cytokines, unsurprisingly, were present at higher levels in samples with more bacteria. Among the exceptions were IL-5 and IL-13, which were positively correlated to bacterial burden in males, but negatively correlated to bacteria in females (**Supplementary Figure 2**, **Supplementary Tables 3, 4**). For another group of cytokines (GRO-α, IP-10, MIP-1α, MIP-1β, IL-27, and LIF), both males and females had positive correlation between cytokine and bacterial burden, but one or both regression coefficients were different between sexes. These sex differences were largely due to females having higher cytokine levels than males in lungs with low bacterial burden. Variation within the population of low-burden samples is interesting, because this is a population that has been challenged, has controlled the bacterial growth usually seen in the preinflammatory phase, but still might succumb depending on the strength of the immune response. IL-5 and IL-13 are associated with Th2 immunity, and GRO-α, IP-10, MIP-1α, and MIP-1β are known to be induced by pattern recognition receptor (PRR) recognition of bacterial pathogen-associated molecular patterns (PAMPs), and produced by neutrophils, being chemotactic for various inflammatory cells (53, 74–76). Supporting the IL-5 and IL-13 findings, we saw that there was a near-significant enhancement (*p* = 0.077) of the eosinophil protein EPO in female low-burden lung samples compared to males, which was not seen for neutrophil protein MPO. Among high-CFU mice, although males had significantly higher bacterial burden, there was no sex difference for MPO or EPO.

The assay best predicting survival in the present study was serological titer against the antigens used in subunit vaccine, as has been seen before (77–79). All vaccinated groups had considerable antibody titers against rF1-V and rV antigens (**Tables 4**, **5**), including those with no protection from mortality or bacterial growth in lungs, but the highest titers were in the groups with the best survival (females given rF1-V prime and LAV boost). Survival was also correlated with titer against TS CO92 whole-cell antigen, though not with titer against TS C12, even though the challenge was with C12. This suggests that the antigens exposed on irradiated whole-cell *Y. pestis* might not reflect the antigens expressed *in vivo* (34). The relatively low antibody titers against whole-cell antigen preparations contrast with substantial *ex vivo* re-stimulation of splenocytes, suggesting that in these preparations there are unspecified molecules that play an important role in cellular immunity. Some of that response is possibly attributed to endotoxin that is present on the LAV and the irradiated samples used for restimulation assays, although *Y. pestis* lipopolysaccharides when grown at 37°C are known to be less stimulatory than endotoxin from other bacteria (80–82).

In comparing the two LAVs, mice vaccinated with P:rF1-V B:Δ*caf1* had greater antibody titers against both TS CO92 and TS C12 than mice vaccinated with P:rF1-V B:Δ*yopD*/Δ*caf1* (**Table 6**). Female mice vaccinated with P:rF1-V B:Δ*caf1* also had higher anti-rF1-V antibody titers than those given P:rF1-V B:Δ*yopD*/Δ*caf1* (**Table 4**). Although the two LAVs were similarly protective, the Δ*yopD*/Δ*caf1* strain might be less immunogenic if it replicates less than Δ*caf1 in vivo* and/or is cleared faster from the body, which was suggested by earlier work showing fitness impairment in *yopD* mutants (46, 83).

We have sought to reduce the Th2 polarization of vaccine-induced immune responses, and make them more balanced between Th1 and Th2, by adding CpG to subunit vaccine formulations (22). This objective is based on the expectation that a robust Th1 response is needed to protect against pneumonic plague (47, 84). In the present study, IgG1 titer measurements were consistently higher than IgG2a, partially due to more sensitive assay conditions, but rF1-V prime/LAV boost led to a more balanced response, with more IgG2a antibodies (linked to Th1) compared to the reverse strategy with LAV prime/rF1-V boost. Similar results were obtained in an earlier study where subunit prime with LAV boost led to more Th1-skewed response, and LAV prime with subunit boost led to more Th2, although in that study all mice survived intranasal challenge (69). We also noted that although the P:Δ*caf1* B:rF1-V regimen induced higher anti-rF1-V titers than P:Δ*yopD*/Δ*caf1* B:rF1-V, the IgG2a response was more pronounced in the latter, with a higher IgG2a/IgG1 ratio in both males and females. The relatively similar IgG1/IgG2a ratios in male and female mice given P:rF1-V B:Δ*yopD*/Δ*caf1* may relate to the similarly high protection in males and females, whereas in mice vaccinated with P:rF1-V B:Δ*caf1*, only 50% protection was achieved in males. A way to further increase the Th1/Th2 ratio could be by administering the vaccine intranasally, which has been seen to produce Th1/Th2 balanced immunity to *Y. pestis*, compared to Th2 bias from subcutaneous immunization (85). Based on other pathogens this could better establish multifunctional and long-lasting CD4 cells in the lungs (86, 87).

Like antibody titers, the number of B cells reacting to *ex vivo* stimulation with *Y. pestis* antigens as detected by ELISpot was higher in mice vaccinated with Δ*caf1* regimens than corresponding groups vaccinated with Δ*yopD*/Δ*caf1*, especially against whole cell antigens. These trends were not seen in T cell ELISpot assays. However, there was some role for cellular immunity in the differential protection between vaccine groups, as seen by differences in the amounts of cytokines secreted upon restimulation.

In splenocytes from vaccinated mice stimulated *ex vivo* with rF1-V, the females immunized with rF1-V prime and LAV boost had less production of cytokines compared to other vaccinated groups (**Supplementary Table 7**). This includes IL-18, IFN-γ, IL-6, MIP-1α, MIP-1β, MCP-3, and IP-10, a suite of cytokines that *in vivo* would be highly inflammatory and attract phagocytes to the tissue (88). Meanwhile, when splenocytes were stimulated *ex vivo* with TS C12, females immunized with P:rF1-V B:Δ*caf1* also had a more limited expression of cytokines compared to the other Δ*caf1* vaccinated groups, with several cytokines underexpressed compared to the PBS group. In females given P:rF1-V B:Δ*yopD*/Δ*caf1*, this pattern was not as noticeable; instead, both males and females had lower expression of several cytokines compared to those given P:Δ*yopD*/Δ*caf1* B:rF1-V (**Supplementary Table 10**). This suggests that more protective vaccines led to less of the damaging systemic inflammation, similar to what we have observed previously in tissue extracts (34). Female mice four weeks post-boost also had a higher percentage of CD44+ splenic CD4 and CD8 T cells than the corresponding males, suggesting more memory cells. A recent study used three common mouse strains and found strain-specific sex differences in immune cell populations at steady state, with BALB/c females notably having more splenic CD8+ T cells, regulatory CD4+ T cells, plasmacytoid DCs, and fewer NK cells than males (89).

### 3.2 Discussion of sex differences in immunity

Sex differences in intrinsic *Y. pestis* resistance have rarely been observed, with most studies showing similar susceptibility between sexes. Several research groups have combined males and females in pneumonic plague experiments without noting any difference in outcomes, using BALB/c mice (78), C57BL/6 mice (49, 90), rats (91), and NHPs (92). In some studies females even fare worse, as assessed by mortality in rats (90) or other markers, such as earlier time to bacteremia in Swiss Webster mice (93). One group looked at Brown Norway rats and saw no difference in mortality but reported minor differences in vital signs as monitored by telemetry (94). In an intravenous infection model using KIM5 and KIM27 strains, naïve males were more susceptible (95–97). But in murine aerosol and intranasal *Y. pestis* infections, clear examples of naïve males being more susceptible to challenge than naïve females were not identified; rather, immunized male mice may be less protected than immunized females.

One study used four strains of mice and found that males vaccinated with rF1 and rV subunits had no impairment in antibody titers, but slightly increased mortality when challenged with the GB strain of *Y. pestis*. The increased mortality was observed in CBA and CB6F1 mouse strains but not in BALB/c or C57BL/6 (98). A more recent study showed surprising differences between males and females vaccinated with rF1-V + Alhydrogel, with vaccinated female BALB/c and C57BL/6 mice being significantly more resistant to challenge than vaccinated males, despite being similar for all immune factors measured in the study (47). Likewise in our current study, we also saw females more likely to be protected by vaccination, most notably vaccination with rF1-V prime and Δ*caf1* LAV boost (100% protection in females and 50% in males in Experiment B, 90% and 60% in Experiment D), and the combination of LAV prime, rF1-V boost, and post-challenge streptomycin (70-90% protection in females and 12-30% in males).

Many sex differences in immune responses to vaccines have been described, whether in mice or human patients. This difference is most often linked to the fact that females have higher antibody levels, both baseline and antigen-specific antibodies induced by vaccines, but also the large number of immune genes and microRNAs on the X chromosome (99). Enhanced humoral immunity protects against infections, but also predisposes females to autoimmune disorders (100). Sex hormones, especially estradiol, influence factors like cytokine secretion, phagocytosis, antigen presentation, and endothelial permeability, with effects varying across different systems (101, 102). Androgens appear to be protective against overactive Th2 immunity in the lungs, as shown in allergy models where female mice had higher levels of eosinophils and associated airway inflammation (103, 104). In this study we saw significantly elevated IL-5 and IL-13 in the lungs of females, compared to males with similar bacterial burden, and EPO in lung tissue was also elevated in females, approaching significance.

The tendency for females to have elevated humoral immune responses does not extend to innate immune factors. Even though experimental injection of endotoxin in women induces more cytokines than in men (105), and female cells express more innate pathogen sensors like TLR2, TLR4 and TLR7 (106, 107), females tend to have innate regulatory mechanisms producing relatively restrained complement activation, controlled proinflammatory cytokine secretion (“cytokine storm”), and better survival during sepsis (56, 57, 108). In one study, male mice had more inflammation and tissue damage in both bacterial and sterile peritonitis, due to differences in resident immune cell populations (107). On the other hand, restrained innate immunity makes female mice more susceptible in some bacterial infection models (109, 110), and women seemingly more susceptible to diseases like cystic fibrosis, listeriosis, and extrapulmonary tuberculosis (111–113).

In pneumonic plague, the phenomenon of the pre-inflammatory phase of infection means that vaccination needs to activate immunity and neutralize bacteria before they can use their immune evasion factors to replicate undetected. An influx of neutrophils is beneficial at this stage, restricting the bacterial growth that would induce a greater, more damaging neutrophil influx later (54, 114). Macrophages and neutrophils derived from female subjects have enhanced phagocytosis in various experimental systems (107, 115, 116), and enhanced bacterial killing by alveolar macrophages (117). Finally, female hormones are well established as beneficial in clearing bacteria from lungs and recovering lung function after injury (118–120).

### 3.3 Interpretation and future directions

Although sex differences in immunity do not appear to produce differences in pneumonic plague survival for naïve animals, we hypothesize that in immunized mice with high opsonizing antibody titer against *Y. pestis*, a synergistic effect could give females more efficient phagocytosis, bacterial killing, and/or cytokine production by phagocytes. This would then lead to earlier influx of neutrophils, minimizing both bacterial spread and the damage of the pro-inflammatory phase. In our highly susceptible mouse model, immunized males had incomplete protection from pneumonic plague, and less beneficial effect from post-challenge antibiotics. This went along with males having lower serum antibody titers, fewer CD44+ T cells, and different cytokine expression patterns, including less IL-5, IL-13, and eosinophil activity in male lungs. Meanwhile, males and females were similar for other factors like IFN-γ secretion by *ex vivo* restimulated T cells, and neutrophil activity in lungs. C3a and C5b-9 levels were higher in males, indicative of higher complement activation in males as seen in previously published data (56, 57). When comparing vaccinated mice to those receiving PBS, vaccinated females had greater reduction in C3a and C5b-9 than vaccinated males did, which may be attributed to lower bacterial load as well as a different antibody response against various epitopes. This may mean that complement activation is enhanced in males, specifically the functional response through third-order avidity interactions with such factors as C1q.

Males and females in our study could all control bacterial spread to some extent, although males who received nonprotective vaccine regimens differed from females by having uncontrolled bacterial replication in the lungs. Further work is needed to establish whether sex differences are seen with other established vaccines and in other models besides mice. It was surprising to us that these sex differences could not be overcome by providing suboptimal post-exposure antibiotic therapy to vaccinated males. We will continue to explore this concept in future experiments.

The immune protection given by our heterologous prime-boost vaccine is likely a combination of enhanced antibody titer against antigens on the bacterial surface; enhanced titer against antigens expressed upon replication; and immune priming that lets innate immune cells respond rapidly without being counteracted by the bacterial Yops that impair phagocyte function so effectively in naïve animals. In addition, the relatively higher IgG2a/IgG1 antibody ratio signifies a stronger T cell response in addition to humoral immunity. Finally, in a heterologous prime-boost incorporating a live attenuated bacterial strain, giving the subunit first would be recommended for safety reasons, as preexisting immunity from the subunit prime should further limit the replication of the LAV (69). Based on survival data, serology, and preferred safety profile, the best vaccine regimen from this study appears to be giving rF1-V with CpG and Alhydrogel as a prime, and *pgm-* pPst-/Δ*caf1* LAV as a boost.

While the sex-related differences reported here are likely species-specific, more work is warranted to further elucidate the sex differences observed in mice vaccinated against plague as they may serve as a unique model to better understand correlates of protection. Working with male mice, particularly under high-containment conditions can be challenging for personnel, and aggression between cagemates can result in other scientific parameters that can confound data (i.e. physical wounding or physiological/immunological differences associated with stress), as recently reviewed by Biltz et al. (121) and Takahashi et al. (122). Social stress differs from other stressors in its effects on the immune system (123), and stress can be beneficial in establishing immune memory and priming innate immunity (124–127) though debilitating long-term (128). In addition to differences between dominant and subordinate mice, subordinate mice are variable in their susceptibility or resilience to a given social stressor (129), and mice wounded during aggressive encounters show immune phenotypes not seen in those that experience stress without wounding (130). These factors lead to greater variance among males than females and are not easily addressed (131). These issues with male mice are significant enough, in our opinion, to justify their exclusion in early exploratory research. However, the results of this study illustrate the importance of testing medical countermeasures using both male and female animals during later-stage research and in advanced development studies before making conclusions that would apply to the entire population.

## 4 Materials and methods

### 4.1 Bacterial strains, media, and growth conditions

The virulent nonencapsulated *Y. pestis* strain C12 was derived from the clinical isolate CO92 (35) through site-directed mutagenesis of the *caf1* gene (36). C12 was used in all challenge experiments. C12 and the encapsulated strain CO92, both inactivated by γ-radiation, were used as antigen stimuli in *in vitro* experiments. CO92 Δ*yscN* and C12 Δ*yscN* were derived from an in-frame deletion of the gene in the CO92 background, as previously described (39). CO92 *pgm*- pPst- is a highly attenuated strain made by curing the pPst plasmid via serial passage at 4°C, and additional passage on Congo Red agar leading to a deletion of the unstable *pgm* locus (39, 41). CO92 *pgm*- pPst- was used as the basis for the novel Δ*caf1* and Δ*yopD*/Δ*caf1* mutants described below.

Bacteria were plated on 5% sheep blood agar (SBA) plates, or tryptose blood agar (TBA) slants, and grown in liquid culture in heart infusion broth supplemented with 0.2% xylose (HIBX). For vaccine injections, bacteria were resuspended in 10 mM potassium phosphate, pH 7.3–7.4 (“KPhos”). SBA was from Remel Inc. (Lenexa, KS); TBA and HIB were from BD Difco (Sparks, MD).

### 4.2 Mutant construction

Primers and plasmids used to construct the novel mutants described herein are listed in **Supplementary Tables 1, 2**. For construction of the *caf1* deletion, flanking regions of the gene were amplified using the primers listed to create a 919 bp upstream fragment and a 973 bp downstream fragment. Overlapping PCR was used to splice the two amplicons together to generate a fragment lacking the coding sequence of *caf1*. The *Y. pestis* fragment contained *Sph*I and *Sac*I restriction sites engineered into the primers and were used to ligate the fragment into the *Y. pestis* suicide vector pCVD442 (132) to generate the plasmid pCVD422-Δ*caf1*. To generate the *Y. pestis* Δ*yopD* mutant, FastCloning method was utilized (133). The Δ*yopD* upstream deletion fragment was 1,053 bp and downstream fragment was 1,038 bps and cloned into pCVD422 plasmid to generate pCVD422-Δ*yopD*. The resulting plasmids were verified by PCR screening and Sanger sequencing.

The pCVD422-Δ*caf1* was electroporated into electrocompetent *Y. pestis pgm-* pPst- (41) using the methods previously described (134) to generate a cointegrate strain. Briefly, cointegrates were selected on LB Lennox agar plates containing 50 μg ampicillin/mL. The cointegrate strains were grown overnight in HIB and plated on LB agar plates containing 5% sucrose to select for allelic exchange recombinants. Those colonies that grew in the presence of sucrose were screened for loss of the *caf1* gene by PCR with the primer pairs listed in **Supplementary Table 2**. To construct the double mutant strain, the pCVD422-Δ*yopD* plasmid was moved into the electrocompetent derived Δ*caf1 Y. pestis* mutant. The allelic exchange mutant was obtained as described above. The Δ*yopD* mutation in this background was confirmed by PCR with the screening primers listed in **Supplementary Table 2**.

The mutants constructed contain several highly stable mutations resulting in attenuation and in some cases exclusion from the US Federal select-agent regulations. The *pgm* deletion was the result of a loss of an approximately 102-kb deletion of the *pgm* locus as described by Jenkins et al. (41). The pPst deletion was the result of curing the entire plasmid from the strain as described by Welkos et al. (135). While the original *caf1* mutant (strain C12 used for challenge experiments) was created by site directed mutagenesis in the fully virulent CO92 strain (36), the *caf1* and *yopD* deletion mutants constructed in the *pgm*- pPst- parental background in this current study resulted from in-frame deletions of the *caf1* and *yopD* genes. The *caf1* deletion retained only the first three nucleotides and last three nucleotides of the coding region and the *yopD* deletion retained only the first 6 nucleotides and last three nucleotides of the coding region. Thus, the mutations created in the respective vaccine strains are very stable and the fact that these strains contain multiple significant mutations make spontaneous reversion highly unlikely if not impossible. If these LAV strains are selected for advanced development, additional phenotypic characterizations and whole-genome sequencing will be carried out.

### 4.3 Animals and immunizations

BALB/c mice (males and females) were purchased from Charles River Laboratories (Frederick, MD), and were 7-9 weeks of age at the time of initial vaccination. Mice were housed in groups of up to 10 in ventilated filter-top cages connected to a common water supply. In some cases, male mice were housed in smaller groups to limit aggressive behavior. Euthanasia was carried out in accordance with IACUC approved early endpoint euthanasia criteria.

### 4.4 Vaccine formulations and immunizations

All vaccine formulations are described in **Table 1**. In this study, all immunizations followed a prime-boost strategy, with two doses of vaccine or sham administered 3-4 weeks apart. Vaccine injections were either LAV (CO92 Δ*yscN*, C12 Δ*yscN*, CO92 *pgm*- pPst- Δ*caf1*, CO92 *pgm*- pPst- Δ*yopD*/Δ*caf1*), or subunit (recombinant F1-V fusion protein [rF1-V] mixed with adjuvant). rF1-V was, formulated under CGMP regulations and stored at -80°C in single-use aliquots (136, 137). Subunit vaccine doses included 2 µg of rF1-V in PBS, mixed with 250 µg Alhydrogel (InvivoGen, San Diego, CA), and 5 µg CpG (ODN 2006, InvivoGen) as additional adjuvant (22). The subunit vaccinations were delivered in 0.1 mL subcutaneous injections. Since all rF1-V subunit formulations were mixed with CpG and Alhydrogel, for the sake of brevity we abbreviate “rF1-V+CpG+Alhydrogel” as “rF1-V”.

For preparation of LAV strains, flasks of HIBX (supplemented with 2.5mM CaCl_2_) were inoculated with a suspension of colonies from a freshly inoculated SBA plate and incubated for 24 h at 28-30°C with shaking (200 rpm). The next day, bacterial concentration was determined by OD_600_, followed by diluting to an OD_600_ of 0.1 into fresh medium and growth for 6 h, then harvesting and resuspending in KPhos to 5.0x10^7^ cells/mL, for a target of approximately 10^7^ cells in each subcutaneously injected dose of 0.2 mL (39). Colony-forming units (CFU) were confirmed by diluting and plating on SBA plates.

### 4.5 Exposure of mice to *Yersinia pestis* challenge

Approximately four weeks after vaccine boost, mice were exposed to challenge doses of aerosolized virulent *Y. pestis* C12. For aerosol doses, colonies from a freshly inoculated TBA slant were suspended in HIBX to an OD_620_ of 0.01 and incubated for approximately 24 h at 28-30°C with shaking (150 rpm). The day of challenge, bacteria were harvested by centrifugation and resuspended in HIB (no supplemental xylose) to a concentration estimated to achieve the desired inhaled dose of bacteria. Exposure to aerosolized bacteria was accomplished as previously described (39). Briefly, mice were temporarily housed in wire mesh cages that were placed in a whole-body aerosol chamber, located within a class 3 biological safety cabinet in a biosafety level-3 laboratory. Aerosols were created by a three-jet Collison nebulizer and delivered to mice for 10 min. Samples of the aerosol were collected in an all-glass impinger, then plated on SBA plates to calculate the bacterial concentration and the estimated inhaled dose (138). When males and females were challenged in the same whole-body aerosolization cohort (**Tables 1**, experiments B and D), age-matched males had higher estimated inhaled doses due to greater minute volumes due to larger body mass (50, 51).

Bacterial burdens in mouse tissue were determined at 3 days post-infection (dpi). After terminal blood collection and euthanasia, spleens and lungs were excised and homogenized in 15 mL Precision tissue grinders (Covidien, Republic of Ireland) in a volume of 1 mL KPhos, while blood was collected in serum separator tubes (365967, BD, San Jose, CA) by terminal collection from axillary vessels. Organ homogenates were diluted in KPhos and plated on SBA plates (100 µl/plate), then CFUs counted. Fresh whole blood was also spread on SBA plates for counting; mice with any CFUs detected in 10 µl blood were considered bacteremic. Limits of detection were 5 CFU/organ and 100 CFU/mL blood. The remainder of the blood was separated, and serum was frozen. All samples from infected mice were sterilized via irradiation with approximately 21 kGy of γ-radiation, and lack of viable bacteria was confirmed by plating on SBA plates before storing samples at -80°C for later use in immunological assays.

### 4.6 Antibiotic administration for layered defense experiments

Streptomycin at 20 mg/kg was delivered via intraperitoneal injection every 6 h for five days. As previously described (34), the antibiotic treatment was initiated at approximately 60 h after exposure to aerosolized *Y. pestis* C12. This resulted in a purposefully sub-optimal antibiotic regimen that could be used to identify synergistic effects between vaccination strategies and antibiotic treatments. Streptomycin was manufactured by XGen Pharmaceuticals (Big Flats, NY) and resuspended in water for injection (Corning Inc., Corning, NY).

### 4.7 Serum antibody measurement

Sera from terminal blood collection were measured for Immunoglobulin G (IgG) antibody levels by semi-quantitative endpoint ELISA in 96-well Immulon 2HB plates (Thermo Fisher, Rochester, NY). Plates were coated overnight with antigens at 4°C as previously described (22, 39). Coating antigen solutions were either rF1-V (2 µg/mL), rV (2 µg/mL), or inactivated *Y. pestis* CO92 or C12 whole cells (10 µg/mL). Recombinant F1-V was stored as described above and V protein (rV) was obtained from BEI Resources (Manassas, VA); aliquots were stored at -80°C until ready to use. *Y. pestis* cells used as antigen had been grown at 28-30°C for 21 h followed by switch to 37°C for an additional 3 h to upregulate the presentation of potential antigens. These antigens were designated TS (temperature-shifted). Two-fold dilutions of serum in PBS/0.05% Tween 20 were made in triplicate and incubated for 30 min at 37°C, then washed and signal detected as previously described (22). Results are reported as the geometric mean (GM) and geometric standard error (GSE) of the reciprocal of the highest dilution giving a mean OD of at least 0.1 ± 1 SD at 450 nm (570 nm used as reference wavelength), then triplicates averaged. The limit of detection was 50.

### 4.8 Splenocyte isolation and ELISpot

Groups of mice were euthanized 25-28 days after the vaccine boost, and splenocytes were isolated using minimal disruption as described previously (39). Briefly, spleens were excised from mice (*n* = 5 mice per group) and disaggregated in RPMI 1640 medium (Gibco, Grand Island, NY). Red blood cells in the spleen homogenate were lysed with Ammonium-Chloride-Potassium (ACK) Lysing Buffer (Gibco) after the extract was diluted with RPMI 1640 medium and cells pelleted by centrifugation at 335 x g for 10 min. Splenocytes were resuspended and counted using a TC20 Cell Counter (Bio-Rad, Hercules, CA) then all samples were diluted in CTL-Test medium (IFN-γ ELISpot) and/or RPMI complete medium (Luminex and B-cell ELISpot) to normalize cell concentrations. Complete medium was RPMI-1640 including 10% fetal bovine serum (HyClone, Logan, UT), 100 U/mL penicillin-streptomycin (Gibco), 1x MEM Non-essential Amino Acid Solution (Sigma, St. Louis, MO) 1 mM sodium pyruvate (Sigma), and 55 µM β-mercaptoethanol (Gibco).

For analysis of cellular responses, splenocytes were incubated in CTL-Test medium (ImmunoSpot, Shaker Heights, OH) and stimulated *in vitro* with rF1-V (25 µg/mL) protein, rV (25 µg/mL) protein, inactivated *Y. pestis* TS CO92 (5 µg/mL), or inactivated *Y. pestis* TS C12 (5 µg/mL) and incubated at 37°C/5% CO_2_. Negative controls were cells stimulated with medium only, while positive controls were stimulated with 100 ng/mL phorbol 12-myristate 13-acetate (PMA) and 500 ng/mL ionomycin. Before cells were plated, plates were coated with capture anti-mouse interferon-gamma (IFN-γ) monoclonal antibody for T-cell ELISpot. After approximately 24 h incubation, ELISpot plates were washed, developed, and spots detected and quantified as previously described (22).

For B cell ELISpot, splenocytes were first stimulated for 5 days with 1x B-Poly S Polyclonal B cell Stimulator (ImmunoSpot) in complete medium. B cell ELISpot plates were coated with rF1-V (15 µg/mL) protein, rV (15 µg/mL) protein, inactivated *Y. pestis* TS CO92 (5 µg/mL), inactivated *Y. pestis* TS C12 (5 µg/mL), or positive control wells coated with Anti-Igk and Anti-Igλ Capture Ab (ImmunoSpot). Antigen-treated plates were washed and splenocytes added and incubated for approximately 8 h, then washed, developed and spots detected as per manufacturer’s instructions.

### 4.9 Cytokine measurement

For mice pre-challenge, splenocytes were isolated and counted as described above, then incubated in RPMI complete medium with antigens rF1-V (25 µg/mL), rV (25 µg/mL), inactivated *Y. pestis* TS CO92 (5 µg/mL), or inactivated *Y. pestis* TS C12 (5 µg/mL) and incubated at 37°C/5% CO_2_. Negative and positive controls were cells stimulated with medium only, or PMA and ionomycin as described above. After approximately 48 h incubation, plates were centrifuged at 1,200 x g for 10 min, then supernatants were harvested for evaluation of cytokine expression.

For mice post-challenge, lung and spleen homogenates at 3 dpi (see above) were frozen at -80°C and irradiated, then later evaluated for measurement of cytokines and other proteins in the tissue. All samples were thawed from cryopreservation, then centrifuged at 10,000 x g for 10 min to minimize debris.

Supernatants and homogenates were measured for cytokine expression levels using the Cytokine & Chemokine 36-Plex Mouse ProcartaPlex panel (Thermo Fisher) and MagPix instrument per manufacturer’s instructions. In each experiment, any cytokine whose standard curve had low R^2^ value (< 0.95) was removed from analysis. Also, cytokines with insufficient positive samples (more than 2/3 of values at or below the lower limit of detection) were removed from analysis. Cytokine levels are expressed in pg/mL.

### 4.10 Protein measurement by ELISA

Lung homogenates at 3 dpi were measured for the proteins eosinophil peroxidase (EPO) and myeloperoxidase (MPO). Homogenates were diluted 1:50 for EPO and 1:500 and 1:5,000 for MPO measurement in the respective assay diluents. Serum samples at 3 dpi were measured for complement proteins C3a and C5b-9, after dilutions of 1:15 and 1:150 (C3a) and 1:50 (C5b-9) in assay diluents. Kits used were: MPO (HK210-02, Hycult, Uden, Netherlands), EPO (CSB-E15830m, Cusabio, Wuhan, China), C3a (NBP2-70037, Novus/Bio-Techne, Minneapolis, MN), and C5b-9 (LS-F22262, LSBio, Seattle, WA).

### 4.11 Splenocyte phenotyping by flow cytometry

For mice pre-challenge, splenocytes were isolated and cryopreserved in freezing medium, then transferred to liquid nitrogen vapor storage. After thawing and counting, cells were labeled with viability dye (L34970 and L34964, Thermo Fisher), washed with FACS buffer, then incubated with 1:200 dilution of Mouse FcBlock (553142, BD Pharmingen, San Diego, CA) in FACS buffer. Cells were stained at 0.5-1.0 x 10^6^ cells/well in a 96-well round-bottom plate. Staining panel used for T cell subsets was CD3e (clone 145-2C11), CD19 (clone 1D3), CD4 (clone GK1.5), CD8a (clone 53-6.7), CD44 (clone IM7), and CD62L (clone MEL-14). Staining panel used for DC subsets was CD3e (clone 145-2C11), CD19 (clone 1D3), CD11c (clone N418), CD11b (clone M1/70), CD8a (clone 53-6.7), CD80 (clone 16-10A1), and CD86 (clone GL1). Samples were fixed in PBS with 2% formaldehyde (Thermo Fisher, Rockford, IL), and run on a FACSCanto II, then analyzed in FlowJo v10.8 (FlowJo, Ashland, OR). FACS buffer was PBS with 1% bovine serum albumin (SH30574, HyClone); freezing medium was 90% fetal bovine serum (SH30396, HyClone) and 10% DMSO (D2660, Sigma).

### 4.12 Statistical analysis

Survival curves of vaccinated and control mice were estimated with the Kaplan-Meier method. Potential synergistic effects of vaccine and antibiotic on survival were analyzed using the Bliss synergy (62). Synergy score values reflect the ratio of median TTD comparing vaccinated and antibiotic treated groups to groups vaccinated without antibiotic and were based on a log-logistic or log-normal parametric survival model.

Correlations were analyzed using the Spearman nonparametric test. Significant differences between groups were compared using the Mann-Whitney test for pairwise comparisons, with relevant comparisons (between males and females in the same vaccine group, or between vaccine groups within the same sex) shown in figures. For linear regressions using bacterial burden to predict cytokine levels, data were ln transformed and slope and intercept calculated for each cytokine, then the method of least squares was used to calculate *p*-values of the difference between females and males for slope or intercept. For ELISA antibody titers, tables show the geometric mean and standard error (GM and GSE) as previously described (34, 39). Values below the limit of detection (LOD) were replaced by LOD/SQRT 2. Statistical analyses were performed using SAS version 9.4, and figures were generated using Prism version 9.4 (GraphPad Software, Boston, MA), except as indicated. Tables were generated using Microsoft Excel. Conditional formatting in tables indicates the range from low (red) to high (green).

## Data availability statement

The raw data supporting the conclusions of this article will be made available by the authors, without undue reservation.

## Ethics statement

The animal research was conducted under an animal use protocol approved by the USAMRIID Institutional Animal Care and Use Committee (IACUC) in compliance with the Animal Welfare Act, PHS Policy, and other Federal statutes and regulations relating to animals and experiments involving animals. The facility where this research was conducted is accredited by the AAALAC International and adheres to principles stated in the Guide for the Care and Use of Laboratory Animals (National Research Council, 2011). 

## Author contributions

MD: Conceptualization, Data curation, Formal analysis, Investigation, Visualization, Writing – original draft, Writing – review & editing. SB: Conceptualization, Data curation, Investigation, Methodology, Resources, Visualization, Writing – original draft, Writing – review & editing. NR: Data curation, Investigation, Writing – review & editing. CK: Data curation, Investigation, Writing – review & editing. MH: Data curation, Investigation, Writing – review & editing. JD: Data curation, Investigation, Writing – review & editing. JM: Investigation, Writing – review & editing. JS: Investigation, Writing – review & editing. KM: Investigation, Writing – review & editing. YT: Investigation, Writing – review & editing. RT: Investigation, Writing – review & editing. JQ: Formal analysis, Visualization, Writing – review & editing. JB: Resources, Investigation, Writing – review & editing, Conceptualization. CC: Formal analysis, Writing – review & editing, Conceptualization, Data curation, Funding acquisition, Investigation, Methodology, Project administration, Resources, Supervision.
