# Supplementary material for: Sex differences in immune protection in mice conferred by heterologous vaccines for pneumonic plague
Source: Front Immunol. 2024 May 21;15:1397579. doi: 10.3389/fimmu.2024.1397579 (PMC11148226; doi:10.3389/fimmu.2024.1397579)

frontiers

Supplementary Material

Sex Differences in Immune Protection in Mice Conferred by Heterologous Vaccines for Pneumonic Plague

Michael L. Davies[1], Sergei S. Biryukov[1], Nathaniel O. Rill[1], Christopher P. Klimko[1], Melissa Hunter[1], Jennifer L. Dankmeyer[1], Jeremy A. Miller[1], Jennifer L. Shoe[1], Kevin D. Mlynek[1], Yuli Talyansky[1], Ronald G. Toothman[1], Ju Qiu[2], Joel A. Bozue[1], Christopher K. Cote[1*]

*** Correspondence:** Christopher K. Cote: christopher.k.cote.civ@health.mil

Supplementary Data

1.1 Supplementary Tables

Supplementary Tables 1-10 are in separate tabs of the accompanying Excel file.

1.2 Supplementary Figures

Supplementary Figure 1. Median lethal dose (LD_{50}) estimations for *Y. pestis* C12 aerosol challenge in male (black) and female (red) BALB/c mice. LD_{50} values were estimated by probit regressions and graphing was performed in SAS version 9.4. The doses delivered to the male mice to calculate the LD_{50} were approximately 2.13×10^3, 1.57×10^4, 3.18×10^5, and 2.50×10^6 inhaled CFUs.

Supplementary Figure 2. Examples of regression lines comparing tissue cytokine level to bacterial burden in male and female lungs. The data in **Table 2** was natural log (ln)-transformed and plotted for linear regression. **(A)** In the regression using CFU/g to predict tissue G-CSF level, regression coefficients for females (F) were intercept: -0.10 and slope: 0.28. Regression coefficients for males (M) were intercept: -0.41 and slope: 0.29, statistically similar to females. **(B)** In the regression using CFU/g to predict tissue IL-13 level, regression coefficients for females were intercept: 3.42 and slope: -0.03. Regression coefficients for males were intercept: 1.60 and slope: 0.05, both significantly different from females based on the method of least squares.

Supplementary Figure 3. Cytokine levels 3 dpi in lungs of mice immunized with Δ*caf1* or Δ*yopD*/Δ*caf1* regimens and challenged with aerosolized *Y. pestis* C12. The seven cytokines whose expression in lungs was not significantly correlated with bacterial burden in **Table 2** were plotted to look for differences between sexes and vaccine groups. **(A)** Cytokine levels were compared across groups of mice given Δ*caf1* vaccine regimens and challenged with aerosolized C12 (4.7×10^5 CFU in females, 5.7×10^5 CFU in males). **(B)** Cytokine levels were compared across groups of mice given Δ*yopD*/Δ*caf1* vaccine regimens and challenged with aerosolized C12 (2.0×10^5 CFU in females, 2.4×10^5 CFU in males). Graphs show each data point and lines representing geometric means. * $p < 0.05$ in Mann-Whitney test comparing males and females in same immunization group.

Supplementary Figure 4. Serum levels of complement proteins in mice vaccinated with Δ*caf1* and Δ*yopD*/Δ*caf1* regimens and challenged with aerosolized *Y. pestis* C12. Three dpi, male (M)

and female (F) mice were euthanized; lung and spleen homogenates were plated to quantify bacteria (CFU/g), and ELISA was used to measure serum samples for the anaphylatoxin C3a **(A-C)** and C5b-9 soluble membrane attack complex **(D-F)**. Serum complement protein level for each mouse was plotted against bacterial burden in lungs **(A,B)** and spleens **(D,E)**. Females and males sham-vaccinated with PBS, and females and males that received any of the heterologous vaccine regimens, were compared for serum levels of C3a **(C)** and C5b-9 **(F)**. ** $p < 0.01$ in Mann-Whitney test.

Supplementary Figure 5. ELISpot assays for IFN-γ-secreting splenocytes from mice given Δ*caf1* and Δ*yopD*/Δ*caf1* vaccine regimens, after *ex vivo* re-stimulation with *Y. pestis* antigens. Four weeks post-boost, female (red) and male (blue) mice were euthanized and splenocytes re-stimulated for 24 h with rF1-V **(A,E)**, rV **(B,F)**, or temperature-shifted whole-cell CO92 **(C,G)** and C12 **(D,H)** antigens, followed by quantification of spots representing cells secreting IFN-γ. Splenocytes from mice given Δ*caf1* vaccine regimens are shown in **A-D**; splenocytes from mice given Δ*yopD*/Δ*caf1* vaccine regimens are shown in **E-H**. Graphs show each data point and lines representing geometric means. * $p < 0.05$, * $p < 0.01$, * $p < 0.001$ in Mann-Whitney test.

Supplementary Figure 6. Sample gating strategy for upregulation of CD44 surface expression. Cryopreserved splenocytes were thawed, incubated with 1:200 Mouse FcBlock, stained with surface antibody cocktail, and fixed in 2% formaldehyde. Samples were run on a FACSCanto II and data analyzed in FlowJo v10.8.

Supplementary Figure 1. **Median lethal dose (LD_{50}) estimations for *Y. pestis* C12 aerosol challenge in male (black) and female (red) BALB/c mice.** LD_{50} values were estimated by probit regressions and graphing was performed in SAS version 9.4. The doses delivered to the male mice to calculate the LD_{50} were approximately $2.13x10^3$, $1.57x10^4$, $3.18x10^5$, and $2.50x10^6$ inhaled CFUs.

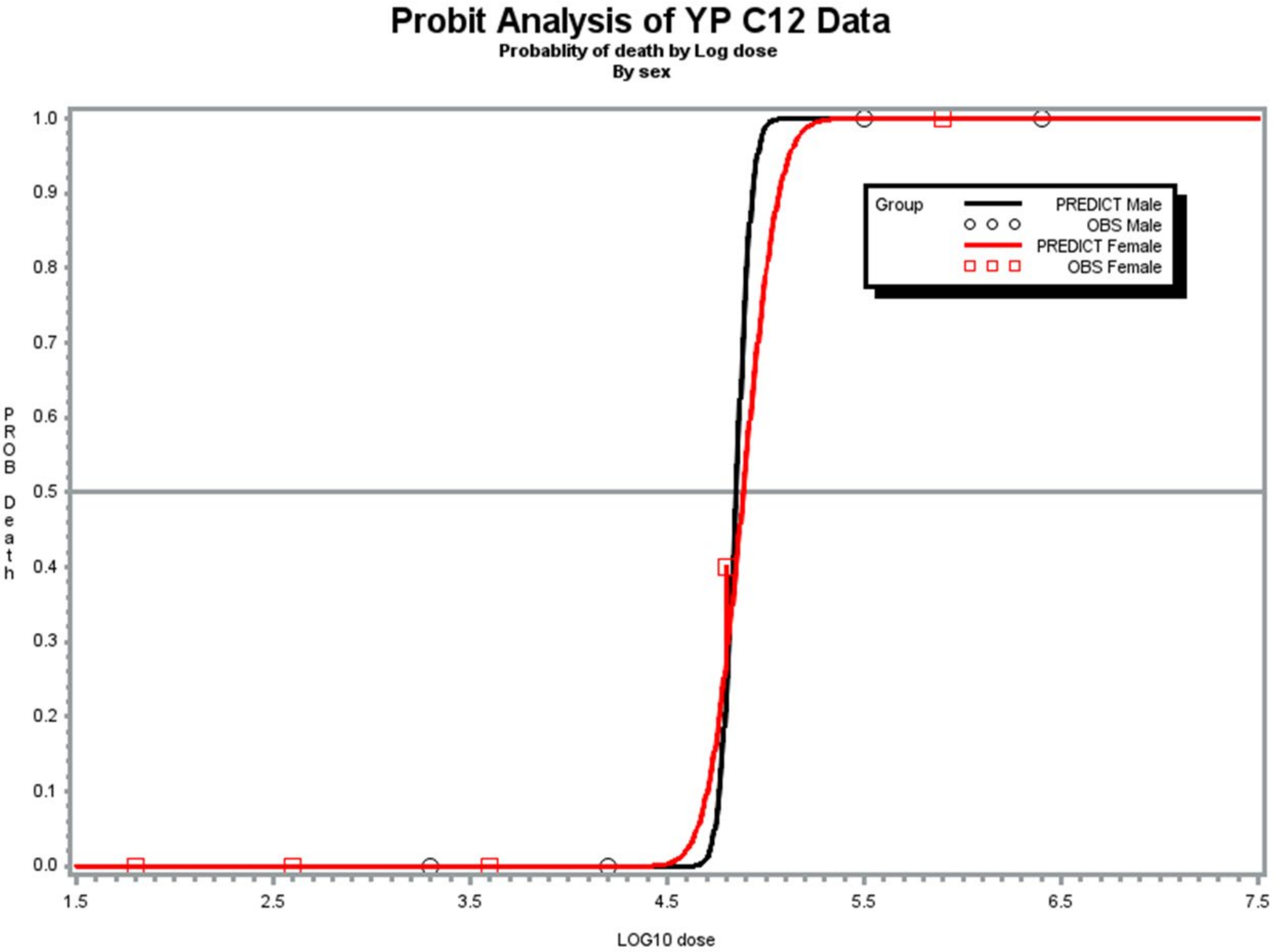

Supplementary Figure 2. Examples of regression lines comparing tissue cytokine level to bacterial burden in male and female lungs. The data in **Table 2** was natural log (ln)-transformed and plotted for linear regression. **(A)** In the regression using CFU/g to predict tissue G-CSF level, regression coefficients for females (F) were intercept: -0.10 and slope: 0.28. Regression coefficients for males (M) were intercept: -0.41 and slope: 0.29, statistically similar to females. **(B)** In the regression using CFU/g to predict tissue IL-13 level, regression coefficients for females were intercept: 3.42 and slope: -0.03. Regression coefficients for males were intercept: 1.60 and slope: 0.05, both significantly different from females based on the method of least squares.

A

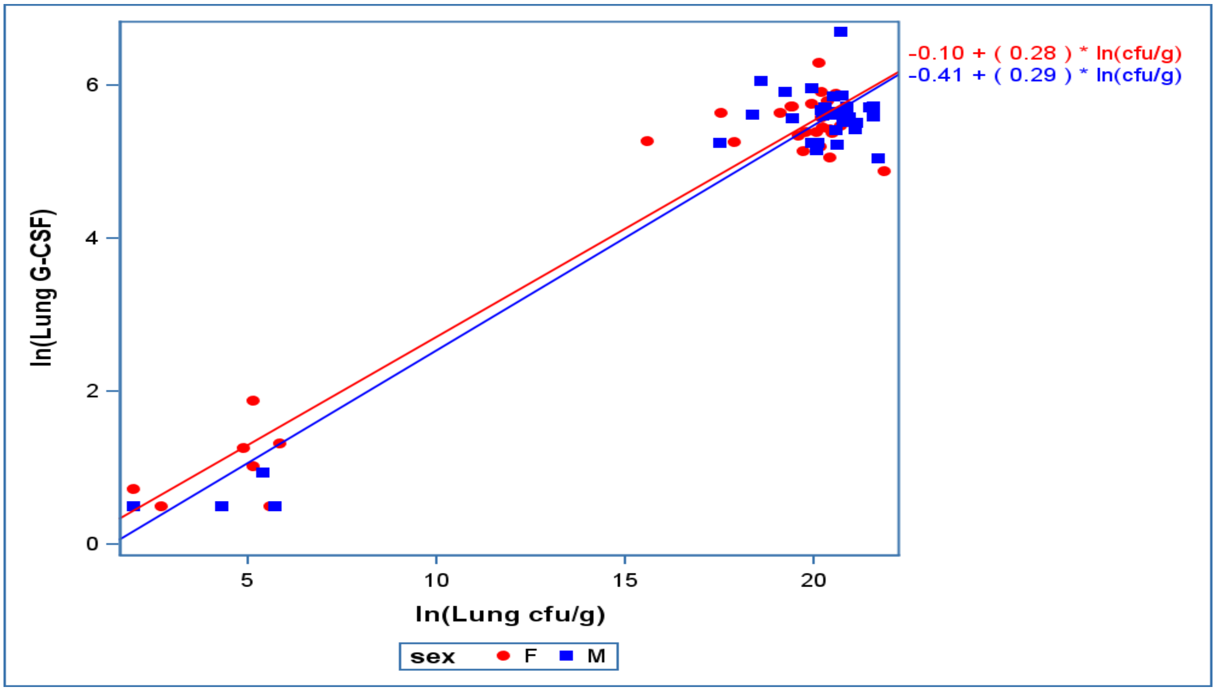

B

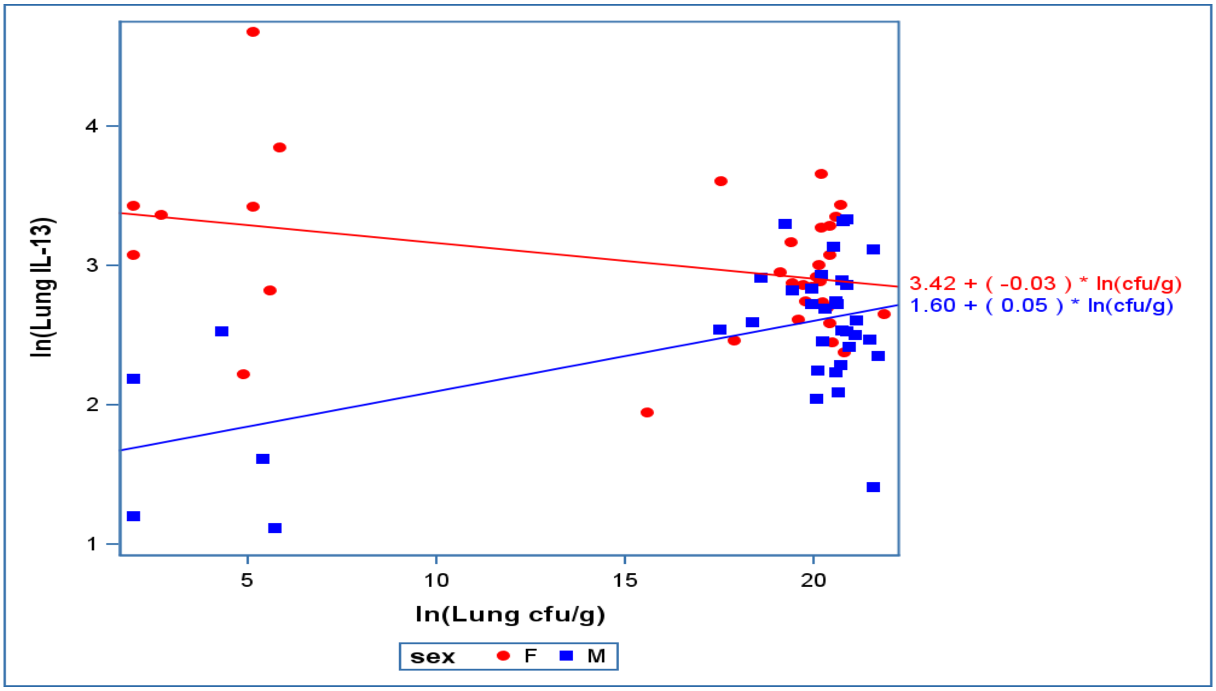

Supplementary Figure 3. Cytokine levels 3 dpi in lungs of mice immunized with Δ*caf1* or Δ*yopD*/Δ*caf1* regimens and challenged with aerosolized *Y. pestis* C12. The seven cytokines whose expression in lungs was not significantly correlated with bacterial burden in **Table 2** were plotted to look for differences between sexes and vaccine groups. **(A)** Cytokine levels were compared across groups of mice given Δ*caf1* vaccine regimens and challenged with aerosolized C12 (4.7 x 10^5 CFU in females, 5.7 x 10^5 CFU in males). **(B)** Cytokine levels were compared across groups of mice given Δ*yopD*/Δ*caf1* vaccine regimens and challenged with aerosolized C12 (2.0 x 10^5 CFU in females, 2.4 x 10^5 CFU in males). Graphs show each data point and lines representing geometric means. * $p < 0.05$ in Mann-Whitney test comparing males and females in same immunization group.

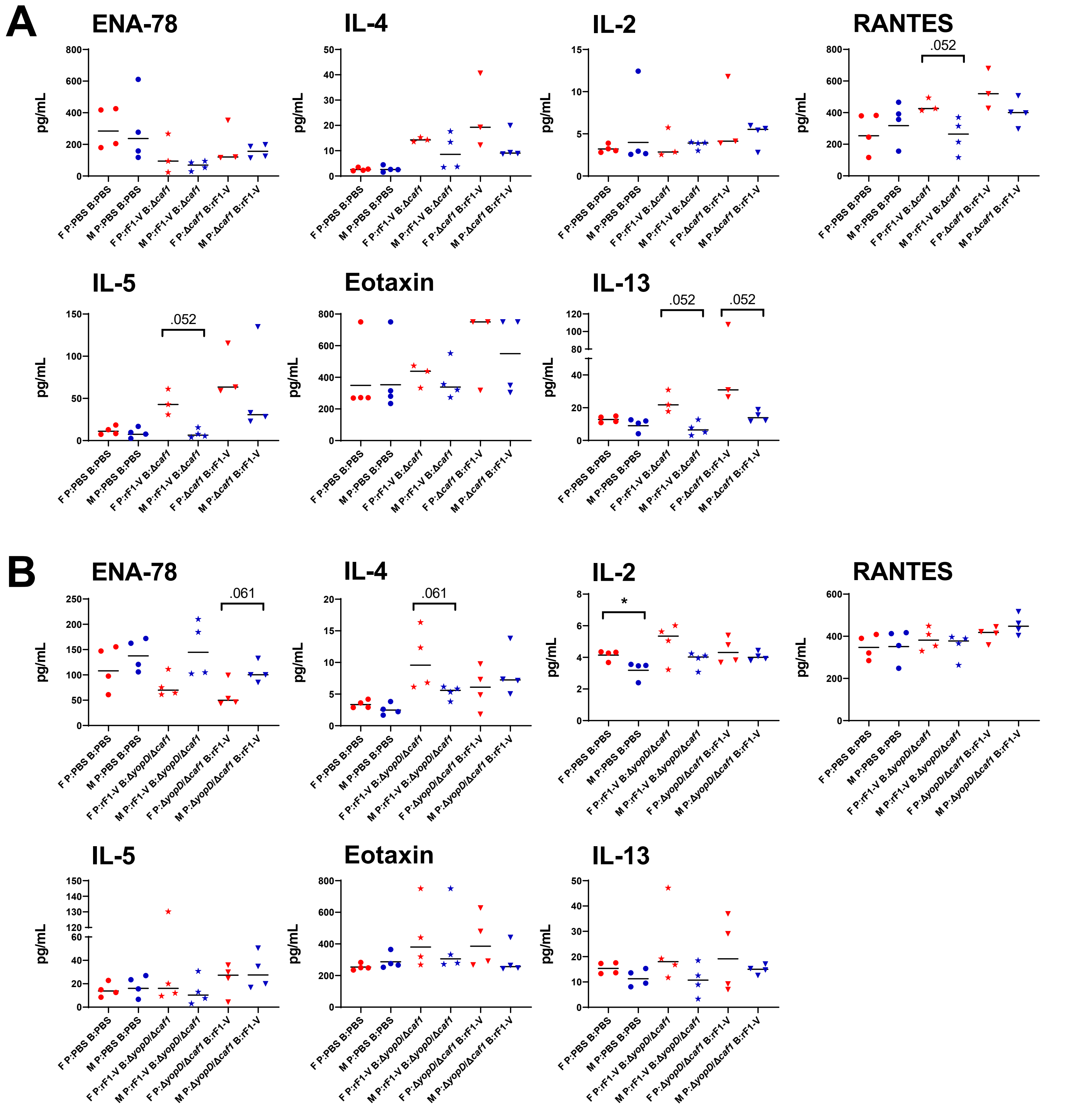

Supplementary Figure 4. Serum levels of complement proteins in mice vaccinated with Δ*caf1* and Δ*yopD*/Δ*caf1* regimens and challenged with aerosolized *Y. pestis* C12. Three dpi, male (M) and female (F) mice were euthanized; lung and spleen homogenates were plated to quantify bacteria (CFU/g), and ELISA was used to measure serum samples for the anaphylatoxin C3a **(A-C)** and C5b-9 soluble membrane attack complex **(D-F)**. Serum complement protein level for each mouse was plotted against bacterial burden in lungs **(A,B)** and spleens **(D,E)**. Females and males sham-vaccinated with PBS, and females and males that received any of the heterologous vaccine regimens, were compared for serum levels of C3a **(C)** and C5b-9 **(F)**. ** $p < 0.01$ in Mann-Whitney test.

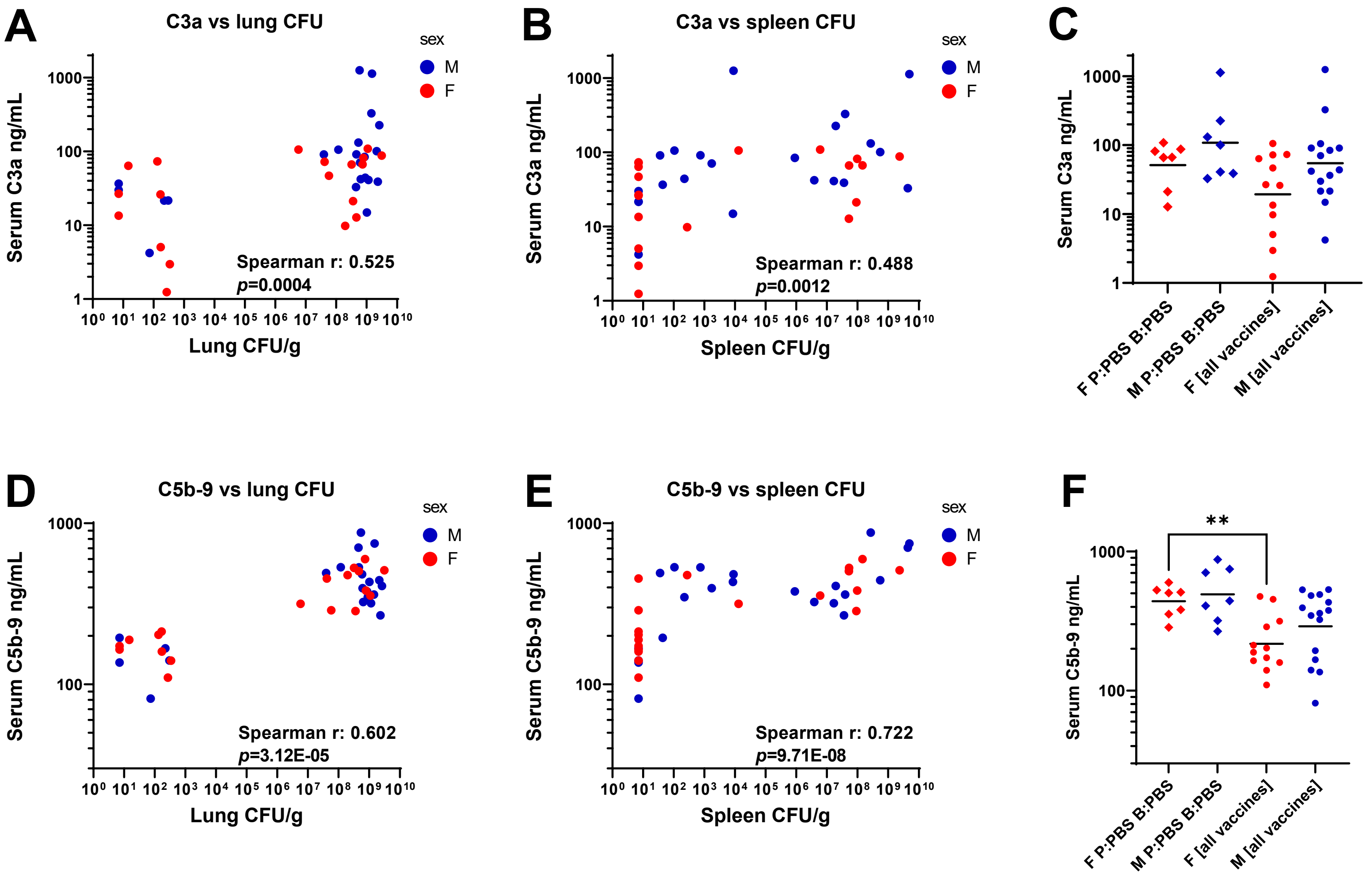

Supplementary Figure 5. ELISpot assays for IFN-γ-secreting splenocytes from mice given Δ*caf1* and Δ*yopD*/Δ*caf1* vaccine regimens, after *ex vivo* re-stimulation with *Y. pestis* antigens. Four weeks post-boost, female (red) and male (blue) mice were euthanized and splenocytes re-stimulated for 24 h with rF1-V **(A,E)**, rV **(B,F)**, or temperature-shifted whole-cell CO92 **(C,G)** and C12 **(D,H)** antigens, followed by quantification of spots representing cells secreting IFN-γ. Splenocytes from mice given Δ*caf1* vaccine regimens are shown in **A-D**; splenocytes from mice given Δ*yopD*/Δ*caf1* vaccine regimens are shown in **E-H**. Graphs show each data point and lines representing geometric means. * $p < 0.05$, * $p < 0.01$, * $p < 0.001$ in Mann-Whitney test.

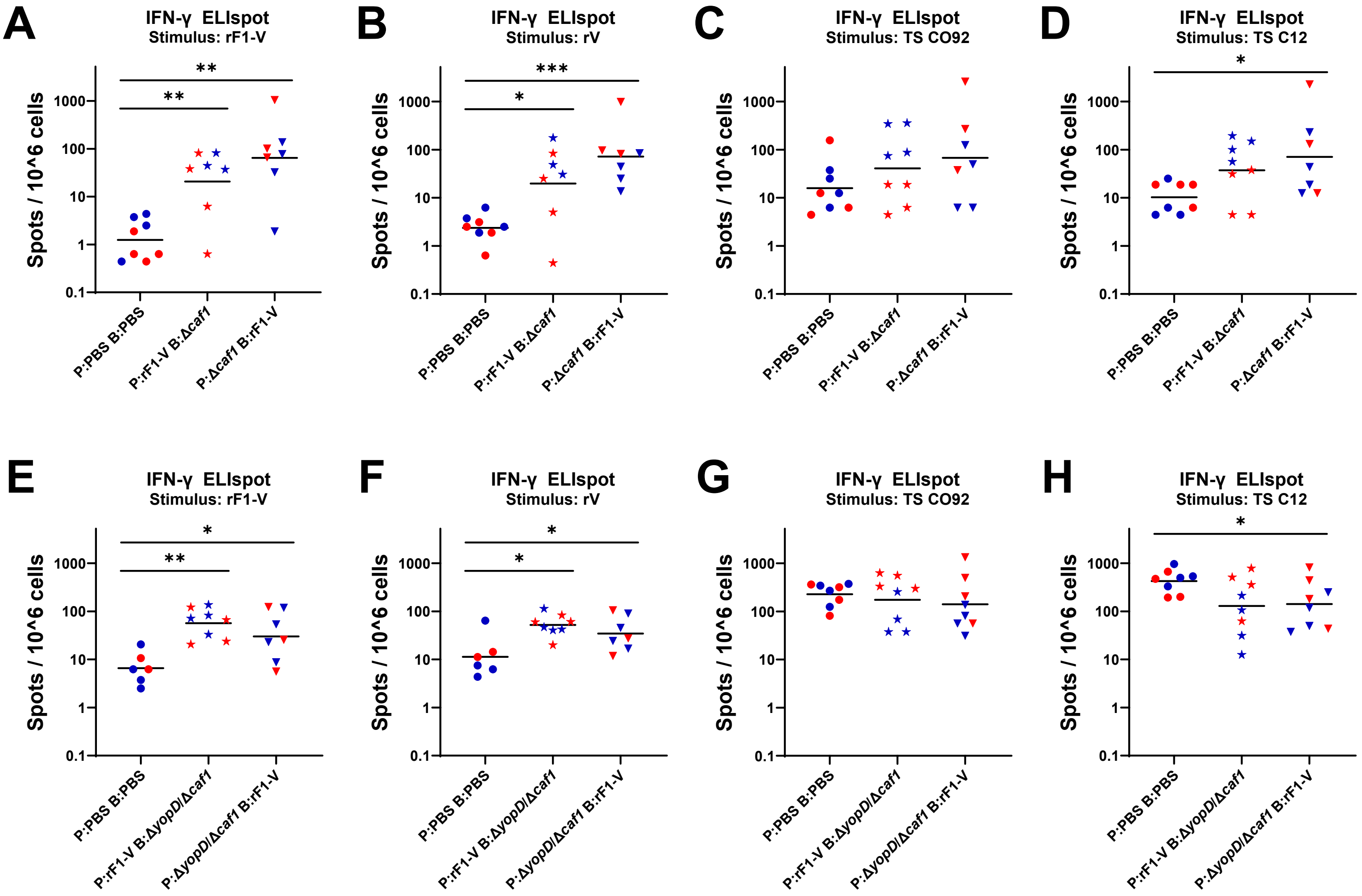

Supplementary Figure 6. Sample gating strategy for upregulation of CD44 surface expression. Cryopreserved splenocytes were thawed, incubated with 1:200 Mouse FcBlock, stained with surface antibody cocktail, and fixed in 2% formaldehyde. Samples were run on a FACSCanto II and data analyzed in FlowJo v10.8.

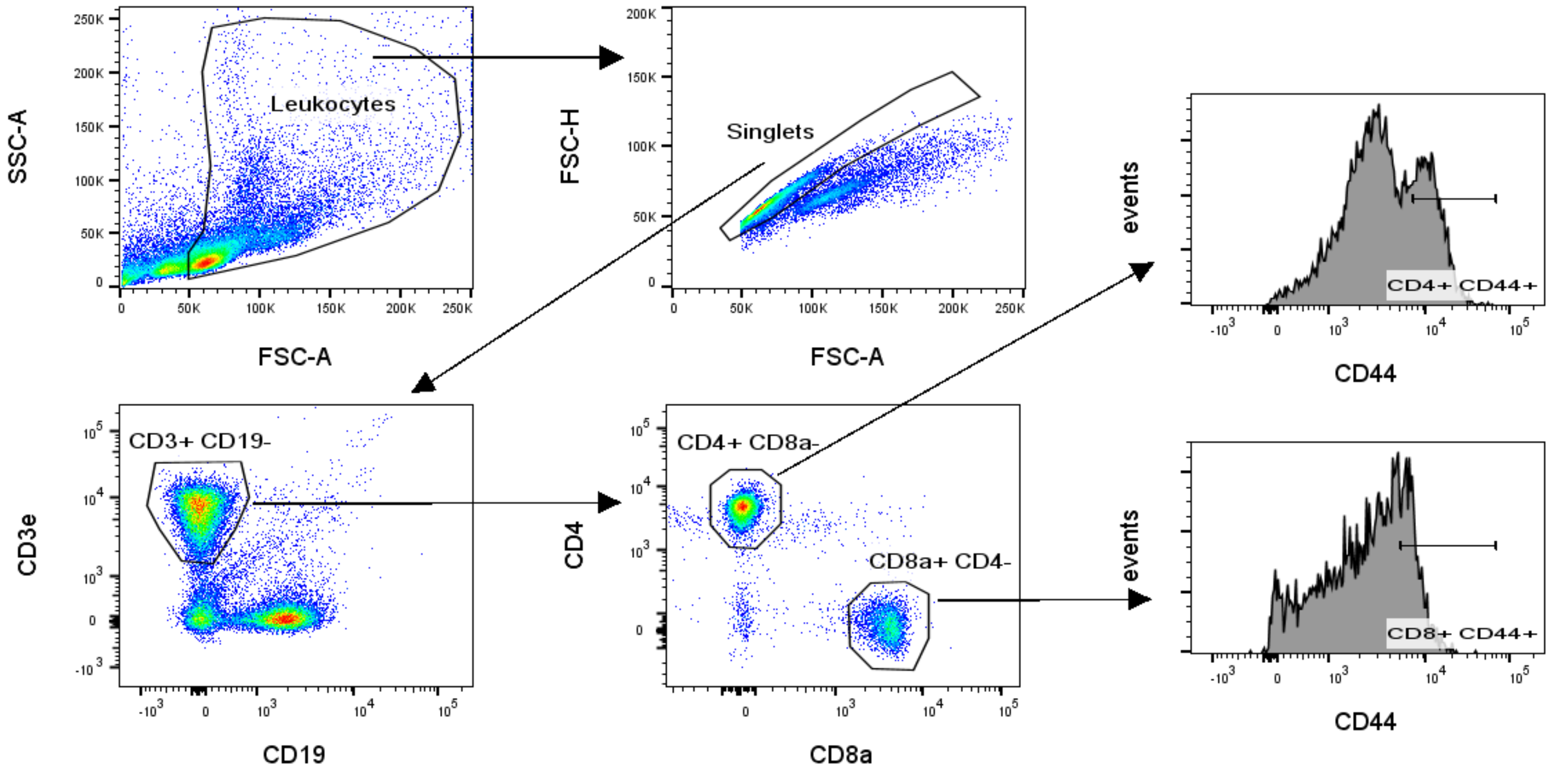

Supplement: Supplementary file 1 [file DataSheet_1.pdf]
